# Effects of Mutations in the *Drosophila melanogaster Rif1* Gene on the Replication and Underreplication of Pericentromeric Heterochromatin in Salivary Gland Polytene Chromosomes

**DOI:** 10.3390/cells9061501

**Published:** 2020-06-19

**Authors:** Tatyana D. Kolesnikova, Alexandra V. Kolodyazhnaya, Galina V. Pokholkova, Veit Schubert, Viktoria V. Dovgan, Svetlana A. Romanenko, Dmitry Yu. Prokopov, Igor F. Zhimulev

**Affiliations:** 1 Institute of Molecular and Cellular Biology, Siberian Branch of Russian Academy of Sciences, 630090 Novosibirsk, Russia; aleksvlako@gmail.com (A.V.K.); galina@mcb.nsc.ru (G.V.P.); dovgan@mcb.nsc.ru (V.V.D.); rosa@mcb.nsc.ru (S.A.R.); dprokopov@mcb.nsc.ru (D.Y.P.); zhimulev@mcb.nsc.ru (I.F.Z.); 2 Laboratory of Structural, Functional and Comparative Genomics, Novosibirsk State University, 630090 Novosibirsk, Russia; 3 Department of Natural Sciences, Novosibirsk State University, 630090 Novosibirsk, Russia; 4 Leibniz Institute of Plant Genetics and Crop Plant Research (IPK) Gatersleben, D-06466 Seeland, Germany; schuberv@ipk-gatersleben.de

**Keywords:** replication timing, *Rif1*, *SuUR*, *Su(var)3-9*, *Drosophila melanogaster*, polytene chromosomes, underreplication, satellite DNA, heterochromatin

## Abstract

In *Drosophila* salivary gland polytene chromosomes, a substantial portion of heterochromatin is underreplicated. The combination of mutations *SuUR^ES^* and *Su(var)3-9^06^* results in the polytenization of a substantial fraction of unique and moderately repeated sequences but has almost no effect on satellite DNA replication. The Rap1 interacting factor 1 (Rif) protein is a conserved regulator of replication timing, and in *Drosophila*, it affects underreplication in polytene chromosomes. We compared the morphology of pericentromeric regions and labeling patterns of in situ hybridization of heterochromatin-specific DNA probes between wild-type salivary gland polytene chromosomes and the chromosomes of *Rif1* mutants and *SuUR Su(var)3-9^06^* double mutants. We show that, despite general similarities, heterochromatin zones exist that are polytenized only in the *Rif1* mutants, and that there are zones that are under specific control of *Su(var)3-9*. In the *Rif1* mutants, we found additional polytenization of the largest blocks of satellite DNA (in particular, satellite 1.688 of chromosome X and simple satellites in chromosomes X and 4) as well as partial polytenization of chromosome Y. Data on pulsed incorporation of 5-ethynyl-2′-deoxyuridine (EdU) into polytene chromosomes indicated that in the *Rif1* mutants, just as in the wild type, most of the heterochromatin becomes replicated during the late S phase. Nevertheless, a significantly increased number of heterochromatin replicons was noted. These results suggest that *Rif1* regulates the activation probability of heterochromatic origins in the satellite DNA region.

## 1. Introduction

The main feature of heterochromatin regions in *Drosophila* polytene chromosomes is underreplication. In the salivary glands polytene chromosomes, underreplication is detectable in the regions of pericentromeric and intercalary heterochromatin [1,2,3,4,5,6]. The underreplication arises primarily due to specific characteristics of the endocycle in these cells. The exit from the S phase occurs before the completion of entire genome replication, and approximately 30% of the genome is underreplicated in polytene chromosomes [7,8,9,10,11,12,13]. The pericentromeric regions, primarily satellite DNA, are underreplicated in the polytene chromosomes because in these regions, late replication origins are localized that do not have sufficient time to activate in the polytene chromosomes because of the truncated S phase. An important feature of intercalary heterochromatin regions is that these are large regions almost devoid of an origin of replication. Replication forks coming from marginal regions do not have sufficient time to complete replication before the end of the S phase [10,14,15,16,17]. The underreplication points to a change in the system of DNA damage control. It is plausible that the intra-S-phase checkpoint and activation of late origins are absent in endocycling nuclei [10,18]. The apoptosis mechanism is turned off in these cells [19]. Genes associated with DNA replication are expressed relatively weakly in endocycling cells [20]. Nevertheless, it has been shown that double-stranded DNA breaks resulting from underreplication can be labeled with antibodies to γH2Av [21] and are partially crosslinked with each other [6], suggesting that the repair system still works in these cells and recognizes these gaps. The repair of double-stranded breaks in pericentromeric heterochromatin, in particular, in satellite DNA clusters, was recently demonstrated directly [22].

In addition to the characteristics of the cell cycle, there are local factors that affect the degree of underreplication. In salivary gland polytene chromosomes, the speed of replication fork movement in the late S phase is significantly lower than that at earlier stages; this phenomenon is unusual for diploid cells [23,24,25]. The factors responsible for such a substantial difference have not yet been clarified, but it can be assumed that proteins Suppressor of UnderReplication (SUUR) and Rap1 interacting factor 1 (Rif1) are involved. It has been reported that in intercalary heterochromatin regions, underreplication is completely suppressed by mutations in the corresponding genes, *SuUR* and *Rif1* [5,26,27]. The effect of the SUUR protein on intercalary heterochromatin is not associated with the emergence of new ORC2-binding sites [28] and is due to the impact on replication fork progression or stability. This notion is evidenced by (1) the ability of SUUR and Rif1 to affect the speed/stability of a replication fork during amplification of chorion genes [27], (2) cell cycle–dependent binding of SUUR to polytene chromosomes [29], and (3) physical interactions of SUUR with PCNA [29] and with CDC45 [30]. Munden et al. [27] have formulated a hypothesis according to which the SUUR protein affects the movement of replication forks during chorion gene amplification and during late replication in salivary gland polytene chromosomes, namely through the targeting of the Rif1 protein to the replication forks, where it attracts PP1 phosphatase. The latter dephosphorylates MCM helicase, which slows down the replication forks [27,31]. A directed mutation in the domain of Rif1 responsible for the binding to PP1 phosphatase completely suppresses underreplication in intercalary heterochromatin [27].

Under the conditions of a histone H1 knockdown, suppression of underreplication in intercalary heterochromatin is observed too [32], and histone H1 acts downstream of SUUR, whereas SUUR acts downstream of Rif1 [27,32].

The intercalary heterochromatin regions correspond to extended domains of tissue-specific genes, that is, they are represented by unique rather than repeated sequences [14,26]. This finding made it possible to obtain detailed underreplication profiles of these areas [6,26,27,32,33]. The presence of intercalary heterochromatin regions leads to approximately ≤19% DNA underrepresentation in euchromatic polytene chromosome arms [6].

The most severe underreplication occurs in pericentromeric heterochromatin. This heterochromatin in *Drosophila* occupies approximately 60 Mb per female haploid genome, i.e., about 33% [34,35]. On the other hand, in the salivary gland polytene chromosomes of 3rd instar wild-type larvae, it makes up only 3% [4]. The study of underreplication in pericentromeric heterochromatin is a challenge because a major proportion of this heterochromatin consists of repeated sequences, which are represented predominantly by satellites and mobile elements. In *D. melanogaster*, over 10 types of simple satellite sequences were identified back in the 1980s, some of which match a consensus sequence, (AAN)n(AN)m [36,37]. Others have larger repeat units: 10 bp [38], 11–12 bp [39] and 359 bp [40]. Modern approaches to the genomic analysis of repetitive sequences have substantially expanded the list of identified heterochromatin tandem repeats [41,42,43,44,45,46,47].

*Drosophila* heterochromatin is molecularly heterogeneous: satellite DNA arrays alternate with more complex sequences that cover individual unique genes or moderate repeats, in particular, mobile elements [34,35,46,47,48,49,50,51]. The level of underreplication differs significantly among various heterochromatin sequences [3,35,52,53,54,55,56,57,58,59,60]. He et al. [61] have revealed a significant correlation between the level of repetition of heterochromatin sequences and the degree of their underreplication. This observation primarily refers to large blocks of satellite DNA and therefore implies a correlation among three factors: the level of underreplication, the size of the satellite block, and the lack of initiation of replication inside these blocks in polytene chromosomes.

According to cytological characteristics, investigators have described (i) α-heterochromatin, which corresponds to a small compact region inside the chromocenter and is virtually unreplicated during polytenization, and (ii) net, diffuse β-heterochromatin, which forms the bulk of the chromocenter of polytene chromosomes [3,4,57,58,59]. β-Heterochromatin forms upon aggregation of fully or partially replicated domains that are physically separated by unreplicated zones [3,35,52,53,54,55,56,57,58,59,60]. β-Heterochromatin morphology can be adopted by both eu- and heterochromatin regions, and there is random variation in the degree of polytenization, depending on the action of modifiers [57,58]. In the salivary gland polytene chromosomes of wild-type larvae, regions proximal to 20B, 39D, 41F, 80A, 81F and 101F have α- and β-heterochromatin morphology [4].

Several factors that affect the underreplication of certain regions of pericentromeric heterochromatin have been discovered. For instance, the underreplication is partially suppressed in *SuUR^ES^* mutants [5,26]. When histone H1 is knocked down, underreplication suppression is also observed in some pericentromeric heterochromatin areas [32]. H3K9 di-/tri-methylation and histone methyltransferase Su(VAR)3-9 affect underreplication of some heterochromatin sequences [33,60,62]. None of these factors has a significant effect on satellite DNA polytenization.

In *SuUR^ES^* mutants, regions with β-heterochromatin morphology acquire a banding pattern [5]. Additional bands are visible in the zones of the eu–heterochromatin transition of all chromosomes. In addition, a large block of mitotic heterochromatin, called Plato Atlantis, appears in chromosome 3 [5,58,59,63,64]. Even more additional material appears in *SuUR^ES^ Su(var)3-9^06^* double mutants [60,62]. For the first time, a clear nomenclature of bands was assigned to regions 20B–20F on chromosome X and regions 40A–F and 41B–D on chromosome 2. For the new material appearing on chromosome 3, a special nomenclature has been introduced: sections PAA–PAF (Plato Atlantis A–F) have been named and described in detail [58,60,63,64].

Recently, it was reported that underreplication in *D. melanogaster* salivary gland polytene chromosomes is presumably fully reversed in *Rif1* mutants [27]. The authors made this assumption on the basis of complete polytenization of all heterochromatin sequences available for genomic analysis in *Rif1* mutants. Furthermore, there was a significant increase in chromocenter size in early 3rd instar larvae. In *D. melanogaster* embryos at the midblastula transition stage, Rif1 binds to (and selectively delays the replication of) large blocks of satellite sequences, and this effect is mediated by the regulation of replication origins [31]. Rif1 inhibits and delays replication initiation by recruiting PP1 phosphatase to the chromosomes where its ability to dephosphorylate kinase targets counters the action of DDK and CDK kinases. Late in the S phase, higher CDK—and likely DDK—concentration causes Rif1 inhibition, which results in origin derepression through pre-RC activation [31]. For polytene chromosomes, the influence of Rif1 on replication timing has been analyzed in detail only for the annotated part of the genome: euchromatin arms and the part of heterochromatin devoid of satellite DNA [27,65]. It has been concluded that Rif1 action is SUUR-dependent and is implemented through the impact on the speed or stability of replication forks [27]. These data indicate the existence of at least two mechanisms of Rif1 action, both requiring the interaction of Rif1 with PP1 phosphatase [27,31]. Of note, a mutation in the *Rif1* gene has an opposite effect on some eu- and heterochromatin regions [65].

*Drosophila SuUR* and *Rif1* mutants are completely viable [5,27,31]. Recent research into the impact of *Rif1* on replication timing in ovarian follicular cells before and after the cells enter the endocycle as well as in diploid cells of larval imaginal discs showed that the removal of functional Rif1 affects only a small fraction of the genome, and this fraction varies in different tissues [65]. It is important to emphasize that in ref. [65], the effect of Rif1 on satellite replication timing was not investigated.

Nevertheless, Rif1 is a conserved protein found in many organisms from yeast to humans. Its functions in the regulation of replication initiation and in the determination of replication timing are conserved too [66,67,68,69]. Therefore, the research into the mechanisms of its action is important and relevant.

In this study, we investigate the effect of mutations in the *Rif1* gene on the pericentromeric heterochromatin of *D. melanogaster* salivary gland polytene chromosomes. We compare this effect with the previously studied effects of mutations *SuUR^ES^* and *Su(var)3-9^06^*. In addition, we examine how mutations in the *Rif1* gene affect pericentromeric-region replication in the polytene chromosomes.

## 2. Materials and Methods

### 2.1. Fly Stocks

Flies were reared on a standard medium at 18 °C, 22 °C or 25 °C. An Oregon R stock served as a wild type. Genotypes of the flies used in this work and descriptions of alleles are given in Table 1. The stocks with mutations *Rif1^1^*, *Rif1^2^* and *Rif1^PP1^* [27] were kindly provided by Jared Nordman. Stocks were constructed in which *In(1)w^m4h^* was combined with chromosomes 2 containing the mutations *Rif1^1^* and *Rif1^2^*. The stock with the *Rif1^KO^* allele [31] was kindly provided by Patrick O’Farrell. The *Suppressor of Underreplication (SuUR^ES^)* mutation was originally detected in an *In(1)sc^V2^* stock [5]. Double-mutant flies *SuUR^ES^ Su(var)3-9^06^* are described in refs [60,62].

### 2.2. Fluorescence in Situ Hybridization (FISH)

DNA clones were labeled with either Tamra-5-dUTP or FLu-12-dUTP (Biosan, Russia) in a random-primed polymerase reaction with a Klenow fragment. A 0.9-kb HindIII fragment of the *28S ribosomal RNA* (*rRNA*) gene [70] and a 1.15-kb BglII fragment from *Stellate* repeats [71] were used. Probes for genes *rpl15* and *CG12406* as well as for heterochromatin-specific variants of 359-bp satellite *1.688Xhet* [72] were amplified and labeled by regular PCR (see Table S1 for details).

To prepare probes that specifically hybridize to satellites with a repeat unit of 5–12 nucleotides, we carried out PCR without a template [73]. For each satellite, optimal PCR conditions were selected experimentally (Table S1). The general program for this type of PCR was as follows: 10 cycles: 94 °C for 1 min, N °C for 15 s and L °C for 40 s; then 30 cycles: 94 °C for 1 min, M °C for 15 s and L °C for 1 min; and the final step: L °C for 5 min (N and M are increasing annealing temperatures and L is extension temperature; they are specific to primers and are shown in Table S1). This PCR usually generated a mixture of products with a length of 100–1000 bp.

These PCR products were employed as templates in labeling PCR. PCR with the same parameters was used for labeling, except that 1 μL (100–200 ng) of the amplicon from the previous reaction was added to the reaction mixture along with 1 µM Tamra-5-dUTP or FLu-12-dUTP (Biosan, Russia).

Salivary glands were dissected in the Ephrussi–Beadle solution, fixed in a 3:1 ethanol/acetic acid mixture overnight at –20 °C, squashed in 45% acetic acid, snap-frozen in liquid nitrogen, and stored in 96% ethanol at –20 °C. Prior to hybridization, the slides were incubated with RNAse A (100 µg/mL in 2× SSC, 30 μL/slide) for 1 h at 37 °C, rehydrated in 2× SSC for 1 h at 60 °C, denatured in 0.07 M NaOH, passed through an ethanol series (70%–80%–96%) for 5 min each and air-dried. Next, 4 μL of a labeled probe was added to 5 μL of water. The probe in water and 1 μL of a calf thymus DNA solution (in different tubes) were heated to 95 °C for 5 min, then were cooled and centrifuged. The probe in water, bovine serum and 20 μL of the hybridization mix (50% formamide, 2× SSC and 10% dextran sulfate) were mixed. The mixture was applied at 15 μL per slide. The hybridization was performed overnight. Hybridization temperatures for individual probes are shown in Table S1. Hybridization temperatures for satellite DNA probes were chosen according to ref. [38] with slight modifications. The unbound probe was removed by three 15-min washes in 0.2× SSC with a gradual increase in temperature (washing temperatures are presented in Table S1). The slides were cooled in PBS (137 mM NaCl, 3 mM KCl, 8 mM NaH_2_PO_4_ and 2 mM KH_2_PO_4_) for 5 min. Next, the squashes were mounted in the VectaShield medium with 0.15 μg/mL DAPI.

### 2.3. Indirect Immunofluorescent Staining

Salivary glands from wandering 3rd instar larvae were dissected in PBS supplemented with 0.1% Tween 20. The glands were then transferred to a formaldehyde-based fixative (0.1 M NaCl, 2 mM KCl, 10 mM NaH_2_PO_4_, 2% NP-40 and 2% formaldehyde) for 1-min incubation. After that, the salivary glands were placed in an acetic acid–formaldehyde mix (45% and 3.2% solutions, respectively) for 1-min incubation and squashed in 45% acetic acid. During immunodetection of histones, the salivary glands were squashed directly in the second fixative, omitting the last 45% acetic acid step. The squashes were snap-frozen in liquid nitrogen, the coverslips were removed and the slides were placed in 70% ethanol and stored at −20 °C.

The slides were washed three times for 5 min in PBS + 0.1% Triton X-100 (PBST). Primary antibodies were incubated overnight at 4 °C in PBST + 0.1% BSA and washed three times with PBST for 5 min each. The primary-antibody dilutions were as follows: a rabbit polyclonal anti-RNAPII Ser5ph (active; phosphorylated at Ser5) antibody (1:200; Active Motif, 39,233) and a mouse monoclonal anti–Phosphorylated Histone H2A Variant (γ-H2AV) antibody (1:1000; Iowa Hybridoma Bank, UNC93-5.2.1) [74].

Secondary antibodies (Alexa Fluor 488- or Alexa Fluor 568–conjugated goat anti-rabbit and anti-mouse IgG antibodies, 1:500; Thermo Fisher Scientific) were incubated for 2 h at room temperature in PBST + 0.1% BSA and washed three times with PBST for 5 min each. The squashes were mounted in the VectaShield medium with 0.15-μg/mL DAPI.

### 2.4. 5-Ethynyl-2′-Deoxyuridine (EdU) Incorporation and Detection

Actively moving wild-type larvae were subjected to an analysis of replication because they have a higher percentage of nuclei in the S phase [29]. Salivary glands were dissected and stored in PBS. EdU incorporation was carried out in a 4 µM EdU solution in PBS. After 10 min EdU incorporation, the salivary glands were moved to PBST for 1-min incubation. The glands were next transferred to a formaldehyde-based fixative (2% NP-40 and 2% formaldehyde in PBS) for 2-min incubation. Then, the salivary glands were placed in an acetic acid–formaldehyde mix (45% and 3.2% solutions, respectively) for 1.5-min incubation and squashed in 45% acetic acid. The squashes were snap-frozen in liquid nitrogen and the coverslips were removed. The slides were stored in 70% ethanol at −20 °C. For EdU detection, the Click-iT™ EdU Alexa Fluor™ 555 Imaging Kit (Thermo Fisher Scientific) was utilized. The slides were first washed in PBS for 20 min, incubated in PBST with BSA for 30 min and then incubated in the reaction cocktail at room temperature for 30 min (40 µL per slide). After triple 5-min washing in PBST, the slides were dried. The squashes were mounted in the VectaShield medium with 0.15 μg/mL DAPI.

For simultaneous detection of replication and transcription, immunodetection of the active form of RNA polymerase II was conducted first, followed by the detection of EdU.

### 2.5. Morphological Analyses and Microscopy of Polytene Chromosomes

Polytene chromosomes were stained with aceto–orcein by standard methods [75]. Briefly, the salivary glands were dissected in 1× PBS, transferred to an aceto–orcein solution (1% orcein in 45% acetic acid) for 10–15 min incubation, followed by incubation in 55% lactic acid for 1–2 min and then were squashed. Phase contrast and fluorescent images were acquired using an Olympus BX51 microscope equipped with a 100×/1.30 Uplan FI Ph3 oil objective and a DP52 camera.

For ultrastructural analysis of polytene chromosomes, the same chromosome fixation protocol was employed as for immunostaining (see above). To analyze replication and the ultrastructure of chromosomes beyond the classic Abbe–Rayleigh limit of ~250 nm, three-dimensional (3D) structured illumination microscopy (3D-SIM) was applied using a Zeiss Elyra PS.1 microscopy system equipped with a Plan-Apochromat 63x/1.4 oil objective and the ZEN Black software (Carl Zeiss GmbH). Image stacks were obtained separately for each fluorochrome using 561 and 405 nm laser lines for excitation with appropriate emission filters [76].

### 2.6. Microdissection

This procedure was performed as described previously [77]. Chromosomal DNA was amplified and labeled by means of WGA kits (Sigma-Aldrich, Saint Louis, MO, USA). Amplified microdissected DNA was used for Illumina sequencing library preparation with TruSeq DNA nano kit (Illumina 20015964) with single indexes. The library was quantified using qPCR Standard curve method with DNA Standards (Kapa Biosystems, KK4903). The libraries were sequenced on Illumina MiSeq instrument as a part of multiplex run.

### 2.7. Data Processing

Adapter sequences and WGA primers were removed using the Cutadapt 1.18 tool [78] with option “--minimum-length 15”. Quality trimming was done using fastp 0.20.0 with the “-l 15” option. Reads were aligned to the *Drosophila* repeated elements library obtained from the Repbase database [79] using [80] blastn 2.9.0 with settings “-perc_identity 80 -qcov_hsp_perc 80 -word_size 7.”

## 3. Results

### 3.1. The Effect of Mutations in the Rif1 Gene on the Morphology of Pericentromeric Regions in Salivary Gland Polytene Chromosomes

**Figure 1:**
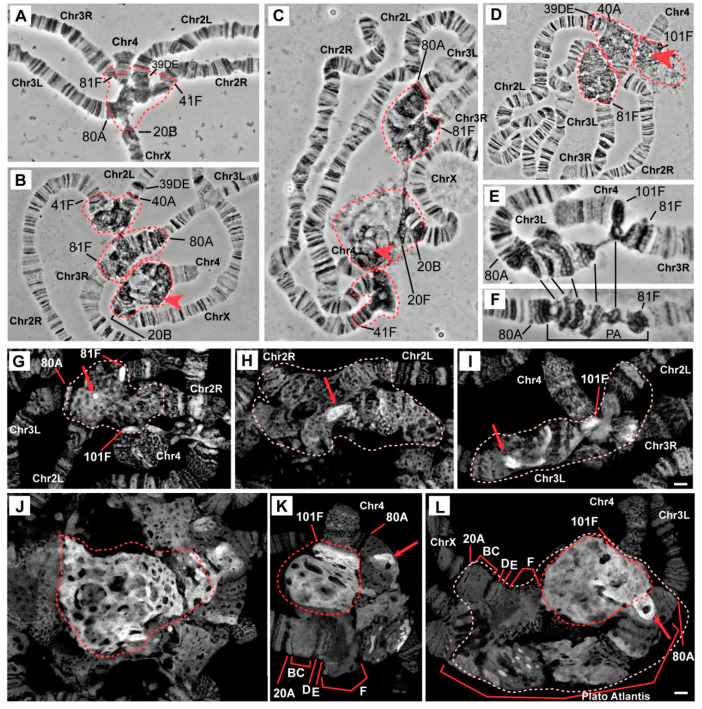
Morphological features of pericentromeric heterochromatin in the polytene chromosomes of *Rif1* mutants. (**A**–**F**) Morphological analysis of the pericentromeric regions of the polytene chromosomes stained with aceto–orcein and examined in phase contrast. (**A**) Wild-type line; pericentromeric regions of all four chromosomes are indicated by a dashed circle. (**B**,**C**) Lines carrying null-allelic mutations in the *Rif1* gene: *Rif1^KO^* [31] (**B**) and *Rif1^1^/Rif1^2^* [27] (**C**); pericentromeric regions of chromosomes 2 and 3 and associations of chromosomes X and 4 are outlined by dashed lines separately. (**D**) Line *Rif1^PP1^*, which carries a directed mutation in the *Rif1* gene that disrupts the binding site for phosphatase PP1 [27]; a red arrowhead in B-D point to a large block of new material appeared at the base of chromosome. (**E**) Pericentromeric region of chromosome 3 (Plato Atlantis) and chromosome 4 in the line with the *Rif1^1^* mutation [27]; (**F**) a pericentromeric region of chromosome 3 (Plato Atlantis) in the *+/Rif1^2^* heterozygous larvae. Lines connect homologous regions of the 3^d^ chromosomes in (**E**,**F**) and illustrate the similarity of all morphological structures. (**G**–**L**) Optical sections of the pericentromeric regions of the polytene chromosomes stained with DAPI (super-resolution 3D-SIM). (**G**) Wild-type line; pericentromeric regions of all four chromosomes are outlined by a dashed line. (**H**) *SuUR^ES^* line; the dashed line delineates the pericentromeric regions of chromosomes 2 and 3. (**I**) The *SuUR^ES^ Su(var)3-9^06^* line; the dashed line indicates the pericentromeric regions of chromosomes 2L, 3L, 3R and 4. (**J**–**L**) Pericentromeric heterochromatin in *Rif1^1^/Rif1^2^* mutants; a dashed line in J delineates a large block of DAPI-positive material; a dashed line in K delineates two discrete zones of DAPI-positive material at the base of chromosome 4; pericentromeric regions of chromosomes X, 3L, 3R and 4 are outlined by a dashed line in L. All visual fields inside polytene nuclei in panels (**G**–**L**) are presented at the same scale. Scale bar 2 µm.

In the salivary gland polytene chromosomes of wild-type larvae, regions proximal to 20B, 40A, 41F, 80A, 81F, and 101F were found to have α- and β-heterochromatin morphology (Figure 1A). Two independent lines carrying mutations in the *Rif1* gene described as null alleles (*Rif1*^−^ (*Rif1^KO^*) [31] and *Rif1^1^/Rif1^2^* [27]) manifest strong and similar changes in the chromocenter morphology in comparison with the wild-type larvae (Figure 1B,C). The most pronounced difference is a general increase in the size of pericentromeric regions of all chromosome arms (indicated by dotted circles in accordance with the dotted line in panel A). A large block of new material appeared at the base of chromosome 4 (marked with a red arrowhead in Figure 1B,C). Chromosome 4 was found to be connected with chromosome X into a single structure in almost all nuclei. The heterochromatin of chromosome 3 is represented by a single well-structured block; there is no break between the left and right arms. The heterochromatin of chromosome 2 was found to be enlarged; in the distal regions, new areas with a banding pattern emerged. All these features were noted in *Rif1^2^* allele homozygotes (data not shown).

In the *Rif1^PP1^* line carrying a directed mutation that disrupts the PP1 phosphatase–binding domain of Rif1 [27], the chromocenter morphology is similar to that of the null mutants (Figure 1D), indicating functional significance of the binding of Rif1 to PP1 phosphatase, i.e., its influence on the underreplication of pericentromeric regions.

In the line with the *Rif1^1^* mutation, also described as the null allele of *Rif1* [27], chromocenter morphology turned out to be unique (Figure 1E). Despite many signs of significant suppression of underreplication in pericentromeric heterochromatin, in particular, the appearance of a large block with a banding pattern between 80A and 81F (similar to Plato Atlantis in *SuUR^ES^* mutants), there are differences with other *Rif1* mutants: (1) between polytene arms 3L and 3R, a zone of underreplication remains, and the arms are connected by a thin thread; (2) the pericentromeric region of chromosome 4 is composed of a slightly enlarged compact material in contrast to the wild type, and chromosome 4 is often associated with the heterochromatin of chromosome 3 and is not attached to chromosome X. The same polytene chromosome morphology was found to be typical for the *Rif1^2^* allele heterozygotes (Figure 1F).

Another feature of the chromocenters in the *Rif1* mutant is the presence of a large amount of DAPI-positive material. Figure 1G–L shows optical sections of pericentromeric regions of the polytene chromosomes in wild-type larvae (Figure 1G) and mutants *SuUR^ES^* (Figure 1H), *SuUR^ES^ Su(var)3-9^06^* (Figure 1I) and *Rif1^1^/Rif1^2^* (Figure 1J–L) fixed with a formaldehyde fixative for maximum structural preservation and stained with DAPI. The analysis of the preparations was performed by super-resolution 3D-SIM microscopy. In the wild-type larvae, pericentromeric heterochromatin is represented by a network structure, where DAPI-positive bands 81F and 101F are distinguishable, which lie at the border of the banded chromosome arms (Figure 1G), and there is only one DAPI-positive spot in the middle of the chromocenter (red arrow in Figure 1G). An analysis of a representative number of nuclei indicated that this spot is likely belongs to chromosome 3 heterochromatin. In mutants *SuUR^ES^* and *SuUR^ES^ Su(var)3-9^06^*, the heterochromatin regions are larger, the material partially acquired a banding structure, and the blocks of the DAPI-positive material became larger and more numerous (Figure 1H,I, red arrows). In contrast, there are huge blocks of DAPI-positive material in *Rif1^1^/Rif1^2^* mutants (Figure 1J, a large block of DAPI-positive material is delineated by a red dotted line). The main contribution to the emergence of a new DAPI-positive material is made by chromatin adjacent to chromosome 4 (Figure 1K,L). It is clearly divided into two zones, where the brightest almost continuous DAPI-positive material adjoins 101F, and behind it, lies less bright more “foamy” material. This format of DAPI-positive material indirectly indicates the polytenization of AT-rich satellite sequences.

Our morphological analysis uncovered additional polytenization in pericentromeric heterochromatin. To find out exactly which sequences underwent additional polytenization, we conducted a series of in situ hybridization assays of heterochromatin sequences from all *D. melanogaster* chromosomes.

### 3.2. Mutations in the Rif1 Gene Result in Polytenization of the Sequences of Proximal Heterochromatin on Chromosome X and Heterochromatin on Chromosome 4

**Figure 2:**
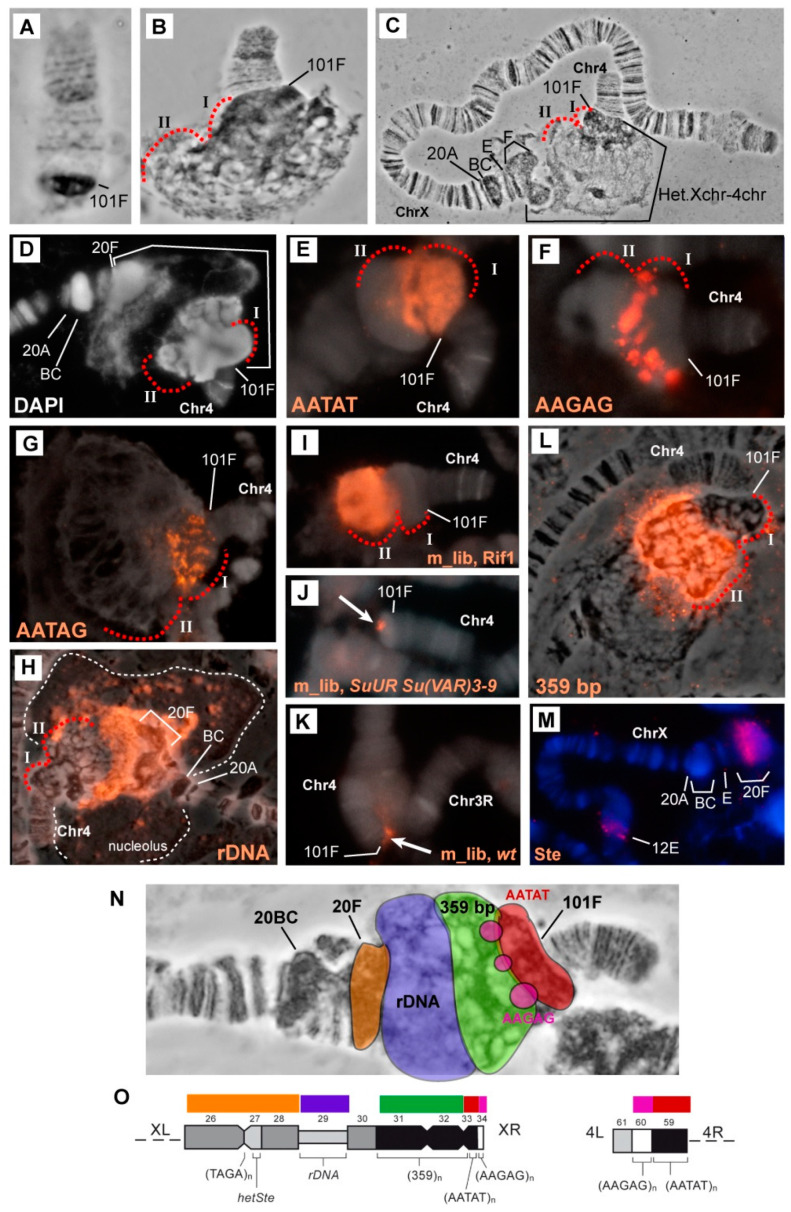
Mutations in the *Rif1* gene lead to the polytenization of satellite sequences from the proximal heterochromatin of chromosomes X and 4 as well as *rRNA* genes. (**A**–**D**) Morphology of the pericentromeric region of chromosome 4 in the control line *SuUR^ES^ Su(var)3-9^06^* (**A**) and in the *Rif1* mutants (**B**–**D**). Red dotted arcs distinguish two zones of different morphology at the base of chromosome 4, indicated by Roman numerals I and II. A merger of the pericentromeric regions of chromosomes X and 4 is indicated by brackets (**C**,**D**). Aceto-orcein staining, phase contrast microscopy (**A**–**C**) and DAPI staining (**D**). (**E**–**M**) In situ hybridization of DNA probes corresponding to heterochromatin sequences from chromosomes X and 4 onto polytene chromosomes of *Rif1^1^/Rif1^2^* (**E**–**I**,**L**,**M**), *SuUR^ES^ Su(var)3-9^06^* (**J**) and wild-type larvae. The probes are indicated in figure; m-lib: microdissection library, the white or blue color: DAPI staining and the red color: a hybridization signal. In all figures, Chr4 denotes chromosome 4, ChrX denotes chromosome X, two zones at the base of chromosome 4 (I and II) are marked with dotted arcs in accordance with panel B. The white outline in H indicates the nucleolus. (**N**,**O**) generalized scheme of localization of all the probes used in relation to the structures of polytene chromosomes X and 4

In polytene chromosomes of wild-type larvae, chromosome 4 usually features a euchromatin part with a small compact block of α-heterochromatin at the base. Mutants *SuUR^ES^* [63] and *SuUR^ES^ Su(var)3-9^06^* (Figure 2A) show a slight increase of this block. In all these genotypes, chromosome 4 tends to come into contact with other heterochromatin regions at both pericentromeric and telomeric ends [81]. At the base of the 4th polytene chromosome of the *Rif1* mutants, the compact chromatin block immediately adjacent to the euchromatin part became significantly larger (Figure 2B). One other block of different structure lies behind it. Because this is new material and there is no nomenclature for it, we designated these two blocks with Roman numerals I and II, respectively. The total amount of new material is comparable in size to the euchromatin part of chromosome 4 (Figure 2B). It turned out that in *Rif1* mutants, polytene chromosomes X and 4 usually form a single structure, uniting with pericentromeric regions (Figure 2C). The morphology of the pericentromeric region 20 of chromosome X in the *Rif1* mutants is similar to that described for *SuUR^ES^* mutants [58,63]: thin band 20A is followed by thick block 20BC, a series of bands 20D–E, and a block of material in 20F, comparable in size to 20BC (Figure 1K,L and Figure 2C). Significant interlinear differences in the morphology of the 20D–F region have been reported [58]. Previously, it has been shown that the *Su(f)* gene is located in region 20E, and all the material in more proximal 20F corresponds to the distal heterochromatin of chromosome X, which is somewhat polytenized in *SuUR^ES^* mutants [35,58,62]. In the *Rif1* mutants, 20F was found to be adjacent to a huge bloating, in which zones of different structures alternate. Differences in structure are clearly visible both in the phase contrast image (Figure 2C) and after staining of the chromosomes with DAPI (Figure 2D): on the side of chromosome 4, two blocks of compact DAPI-positive material are visible, and in the middle of a bulge, there is looser material. Given that all the material lying between 20F and 101F does not resemble any structures previously documented for *D. melanogaster* polytene chromosomes, we performed a series of in situ hybridization assays with probes located in the pericentromeric heterochromatin of chromosomes 4 and X.

**Figure 3:**
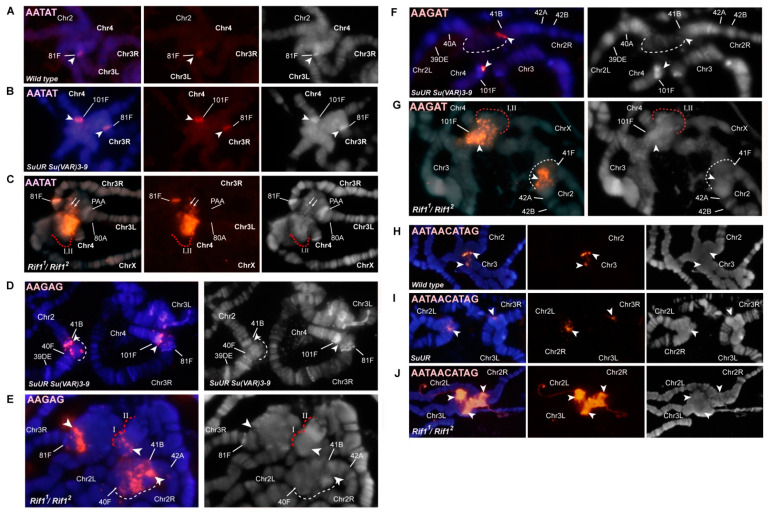
Comparison of the effects of mutations *Rif1^1^/Rif1^2^* and *SuUR^ES^ Su(var)3-9^06^* on polytenization of simple satellites. In situ hybridization of DNA probes specific to satellites AATAT (**A**–**C**), AAGAG (**D**,**E**), AAGAT (**F**,**G**) or AATAACATAG (Prodsat) onto polytene chromosomes of wild-type larvae (**A**,**H**) and mutants *SuUR^ES^ Su(var)3-9^06^* (**B**,**D**,**F**,**I**) and *Rif1^1^/Rif1^2^* (**C**,**E**,**G**,**J**). No signal was detected in wild-type polytene chromosomes with the probes to AATAG and AAGAT satellites under the same hybridization conditions (data not shown). White dashed lines indicate the β-heterochromatin of chromosome 2 and red dashed lines indicate zones II and II at the base of chromosome 4. The left column is a superposition of the hybridization signal and DAPI staining, the right column is the DAPI staining, and the central column in (**A**) is the hybridization signal.

According to the map of chromosome 4 mitotic heterochromatin, the heterochromatin adjacent to euchromatin consists mainly of the AATAT satellite (block h61, Figure 2O). In *Rif1^1^/Rif1^2^* polytene chromosomes, the AATAT satellite signal covers a large compact block adjacent to the euchromatin part of chromosome 4 (Figure 2E). Figure 3 depicts a comparison of the hybridization signals among wild-type polytene chromosomes and *SuUR^ES^ Su(var)3-9^06^* and *Rif1* mutants. In *SuUR^ES^ Su(var)3-9^06^* mutants under the same hybridization conditions, we detected an AATAT signal in a compact block at the base of chromosome 4, but this signal was much smaller and weaker (Figure 3B,C), in accordance with the small size of the corresponding structure. In the wild-type larvae, we either did not see the signal or it merged with the weak signal in the 81F region of chromosome 3 (Figure 3A). This observation suggested that the 4^th^ chromosome block of the AATAT satellite is additionally polytenized in the *Rif1* mutants. 

The next large block of mitotic heterochromatin h60 is represented mainly by the AAGAG satellite. In the polytene chromosomes of the *Rif1* mutants, the AAGAG satellite yielded multiple signal spots in the zone lying farther from the euchromatin part of chromosome 4 than the AATAT block does (Figure 2F and Figure 3E). This structure is absent in *SuUR^ES^ Su(var)3-9^06^* mutants; only a trace signal point can be seen at the base of chromosome 4 (Figure 3D).

Another satellite that was relatively recently located in the heterochromatin of chromosome 4 and proven to be directly adjacent to its centromere is the AAGAT satellite [46]. This satellite gave a sufficiently bright but point signal at the base of chromosome 4 in *SuUR^ES^ Su(var)3-9^06^* mutants (Figure 3F). In the *Rif1* mutants, we noted multiple spot signals interspersed in large block I stained by the AATAT hybridization probe (Figure 2G and Figure 3G). It can be concluded that this satellite has a large number of copies on chromosome 4 and that it may match unannotated block h59.

Thus, all satellites of the AANAN type from chromosome 4 were found to be significantly polytenized in the presence of the *Rif1* mutation (Figure 3A–G).

To find out what the block located behind the AATAT block corresponds to, we performed microdissection of the chromatin material lying at the base of polytene chromosome 4 behind the compact block of the AATAT satellite. For the microdissection, chromosomes were selected that were detached from the total mass of the chromocenter and were positioned as in Figure 2B. Six libraries were prepared that were hybridized in situ. All the libraries yielded a signal in the area selected for microdissection, but only one library gave a clearly specific major signal only at the base of chromosome 4 (Figure 2I). Therefore, this library was chosen for further analyses. Figure 2J shows that in contrast to the *Rif1* mutants, in *SuUR^ES^ Su(var)3-9^06^* double mutants as well as in the chromosomes of wild-type larvae (Figure 2K), this material yielded only a small point signal in the chromocenter, mainly at the base of chromosome 4. That is, this material is under specific control of the Rif1 protein. In addition to the signals in chromosome X, we saw two minor signals in the heterochromatin of chromosome 3.

We performed massively parallel sequencing of the microdissection library on a High-Throughput Illumina MiSeq sequencer. As a result, we obtained 75,505 pairs of reads, with a total length of 8.36 Mb. After the removal of adapter sequences and WGA primers in the Cutadapt 1.18 software (minimum length 15 bp, reads shorter than this length were not retained), 30,691 paired reads with a total length of 2.67 Mb remained. Sequences were quality-filtered using the fastp 0.20.0 tool, after which 27,623 pairs of high-quality reads with a total length of 2.37 Mb were left. Duplicated reads were removed by means of the NGSReadsTreatment tool. The sequences were then aligned to the *Drosophila* Repeated Elements Library retrieved from the Repbase database using blastn. Only alignments with identity and coverage of ≥80% were retained, and 5493 reads aligned with sequences in the library (Table S2).

Of these, 435 reads matched different mobile elements, in the vast majority of cases, retrotransposons; 43 corresponded to rRNAs. This finding makes sense because the microdissected block lies next to ribosomal DNA (rDNA) in polytene chromosome squashes. The remaining reads showed more than 80% homology with the repeats annotated in the database of repeated sequences from five related families of repeats. All these repeats are annotated for the *Drosophila* genome, are located mainly on chromosome X and correspond to a different version of *D. melanogaster* satellite 1.688 (according to the database at https://www.dfam.org [82]). These are families ARS406_DM (2511 reads), SAR2_DM#Satellite (1111 reads), SAR_DM#Satellite (1352 reads) and XDMR # Unknown (58 reads).

Another 31 reads with 80–85% homology to SAT-1_DSim#Satellite represent a satellite related to 1.688 in closely related *Drosophila* species. After a search for corresponding sequences in the *D. melanogaster* genome (6th release), the SAR_DM # Satellite and SAR2_DM#Satellite groups were found, but the homology was ~50%. Thus, we are probably dealing with related sequences that are different versions of satellite 1.688, some of which are not located in annotated genomic scaffolds.

Kim et al. [72] have described a consensus sequence for heterochromatin copies of *D. melanogaster* satellite 1.688. Using this consensus, we designed a pair of primers that specifically amplify heterochromatin copies of this satellite. In silico PCR with these primers (in the UCSC Table Browser) produced ~100 different amplicons, but all of them fall into one of the listed repetition families.

We ran PCR with these primers and performed in situ hybridization of the obtained probe with the polytene chromosomes of *Rif1^1^/Rif1^2^* mutants. We obtained very intense labeling specific to the zone at the base of chromosome 4: exactly where the labeled DNA of the microdissection library hybridized. As in the case of the library hybridization, two minor signals were visible in the heterochromatin of chromosome 3. This pattern fully matches the distribution of the 1.688 satellite in mitotic and polytene chromosomes as described in the literature [44,60,83].

Thus, the structure that in polytene chromosomes looks like a part of chromosome 4 corresponds to the proximal heterochromatin of chromosome X. The blocks of AATAT and AAGAG satellites are located not only in the heterochromatin of the chromosome 4, but also in chromosome X proximally to satellite 1.688. Thus, we cannot determine which of the two chromosomes is responsible for each of the AATAT and AAGAG in situ hybridization signals in this structure.

Simple repeats were not represented in our library, probably because such repeats are always underrepresented in raw reads, that is, they are represented less than expected based on their proportion in the genome [43,44,84].

Next, we hybridized the 28S rDNA probe. In the polytene chromosomes of wild-type larvae, distal heterochromatin (lying between the eu–heterochromatin border and the nucleolar organizer) was found to be not polytenized. Accordingly, in the polytene chromosome squashes, the nucleolus was often not associated with the bulk of chromosome X and is likely a separate structure. In situ hybridization produced a cloud of spots that exactly match the DAPI-positive areas of the nucleolus (Figure S1A). In the study by Belyaeva et al. [5], it was shown that only 4% of rDNA is polytenized in wild-type polytene chromosomes. In *SuUR^ES^* mutants, the level of rDNA polytenization is ~20% [5]. In situ hybridization of the 28S rDNA probe with the polytene chromosomes of the *SuUR^ES^* line revealed that the signal also includes spots located in the DAPI-positive zones of the nucleolus. A zone at 20F was also found to be labeled where SCLR repeats are located that are composed of damaged heterochromatic variants of *Stellate* genes, copia-like elements, LINEs and rDNA fragments [71]. In the *SuUR^ES^* mutant, the nucleolus often does not separate and remains associated with distal heterochromatin (Figure S1B). In the *Rif1* mutants, the 28S rDNA signal is divided into discrete signals in the form of spots in the nucleolus; in addition, we detected a huge solid signal in the DAPI-pale part of the X-4 joint structure (Figure 2H and Figure S1C).

To find out whether rDNA was completely polytenized in the *Rif1* mutants, we stained the *Rif1^1^/Rif1^2^* chromosomes with antibodies to γH2AX, whose distribution in polytene chromosomes correlates with underreplication [21]. We saw that the corresponding zone gives the strongest signal (Figure S2). This observation implied that some underreplication remains in the rDNA cluster.

The distal heterochromatin of chromosome X in mutants *Su(var)3-9^06^* and *SuUR^ES^ Su(var)3-9^06^* is decompacted and forms a giant pseudo-puff [62]. We found that in the *SuUR^ES^* and *Rif1* mutants, such a pseudo-puff does not form and region 20F looks similar. The *Stellate* repeat was located in the distal heterochromatin of chromosome X [47,71] and in polytene chromosome X, it gave an in situ hybridization signal in the 20F region [62]. In line with the literature data, we localized the *Stellate* repeat at 20F in *Rif1^1^/Rif2^2^* polytene chromosomes (Figure 2M).

To summarize the results of hybridization, we present a generalized scheme of localization of all the probes used in relation to the structures of polytene chromosomes X and 4 (Figure 2N,O). We can conclude that all the satellite repeats we tested (satellites 1.688, AATAT and AAGAG) and rDNA repeats are significantly polytenized in the *Rif1* mutants as compared to *SuUR^ES^* and *SuUR^ES^ Su(var)3-9^06^*. On the other hand, according to the literature, mutation *Su(var)3-9^06^* significantly affects replication timing and underreplication of the distal heterochromatin of the X chromosome [62], whereas *SuUR^ES^* and *Rif1* affect this area much less.

### 3.3. In the Rif1 Mutants, Chromosome Y is Visible in Salivary Gland Polytene Chromosomes

The Y chromosome of *D. melanogaster* is completely heterochromatic. It contains ~40 Mb of DNA and accounts for 20% of the male haploid genome [34,35,50]. In the polytene chromosomes of wild-type larvae, the Y chromosome is not polytenized and not visible. It remains invisible in the presence of *SuUR^ES^ Su(var)3-9^06^* mutations [35]. Thus, no one has ever seen the Y chromosome in *Drosophila* salivary gland polytene chromosomes.

**Figure 4:**
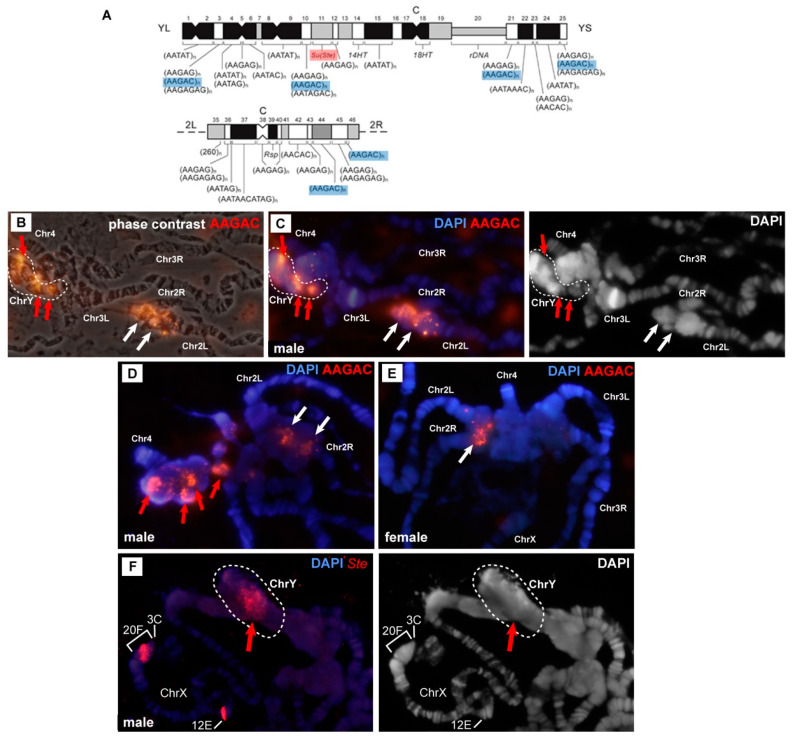
Visualization of the Y chromosome in the polytene chromosomes of the *Rif1* mutants. (**A**) Localization of the AAGAC satellite (marked in blue) and *Su(Ste)* locus (highlighted in red) on the mitotic heterochromatin map (adapted from [35]). (**B**–**D**) In situ hybridization of the AAGAC satellite probe to the polytene chromosomes of male (**B**–**E**) and female (**D**) *Rif1^1^/Rif1^2^* mutants. White arrows point to a signal in the heterochromatin of chromosome 2. Red arrows indicate a signal corresponding to the Y chromosome. (**F**) Hybridization of the *Stellate* probe on the chromosomes of an *In(1)w^m4h^/Y; Rif1^1^/Rif2^2^* male. The red arrow indicates a hybridization signal on chromosome Y. Dashed lines surround the material that is supposedly chromosome Y.

We used a probe for satellite AAGAC, which, according to the mitotic map, is located in the right arm of chromosome 2 and in four DAPI-negative blocks of chromosome Y (Figure 4A). In the male chromosomes, there was a signal in chromosome 2 heterochromatin (Figure 4B–D, white arrows) and additional multiple signals in heterochromatin (Figure 4B, red arrows). The location of the signals varied from nucleus to nucleus, and most often, they were associated with a block of material at the base of chromosome 4 (Figure 4D). In some cases, the signals were confined to a structure that did not correspond to any structures that we saw in the females (Figure 4B,C). Figure 4C presents a structure where DAPI-positive and DAPI-negative zones alternate. DAPI-negative zones turned out to be intensely labeled with the probe to AAGAC. We propose that this structure is the Y chromosome polytenized in the polytene chromosomes of the *Rif1* mutants. In a phase contrast image (Figure 4B), this entity looks like a structureless material, and without additional markers, it cannot be unambiguously detected in the mass of β-heterochromatin. In the female polytene chromosomes of *Rif1^1^/Rif2^2^* larvae, only two signals corresponding to chromosome 2 and no other signals are visible (Figure 4E, white arrow).

To prove the presence of polytene chromosome Y in the *Rif1* mutants, we localized the probe corresponding to *Stellate* repeats on the chromosomes of a male with genotype *In(1)w^m4h^/Y; Rif1^1^/Rif2^2^*. In wild-type and in *SuUR^ES^ Su(var)3-9^06^* polytene chromosomes, the *Stellate* probe hybridized to euchromatin gene *Su(Ste)-like* (*Ssl* or *CK2β*) in region 12E and to the distal heterochromatin (polytene region 20F) of chromosome X [62]. In *D. melanogaster* chromosome Y, the *Suppressor of Stellate [Su(Ste)]* locus is present, which contains more than 600 copies of the *Stellate* repeat [47]. When hybridizing the *Stellate* probe with *In(1)w^m4h^/Y; Rif1^1^/Rif2^2^* polytene chromosomes, we clearly saw two hybridization signals on the X chromosome: one in region 12E, and the other in 20F, which was transferred by an inversion in the middle of the X chromosome. In addition, we saw a prominent signal in the chromocenter (Figure 4F). Most often, the signal was associated with the base of chromosome 4, where new DAPI-positive bands emerged. In rare cases, the signal was visible in a separate structure (Figure 4F). We used the inversion to transfer the heterochromatin X-chromosome *Stellate* repeats away from the chromocenter; therefore, we know that the signal in the chromocenter is not associated with these copies. Our data indicated that in the *Rif1* mutants, chromosome Y is visible in salivary gland polytene chromosomes.

### 3.4. The effect of Rif1 on the Heterochromatin of Chromosome 2

**Figure 5:**
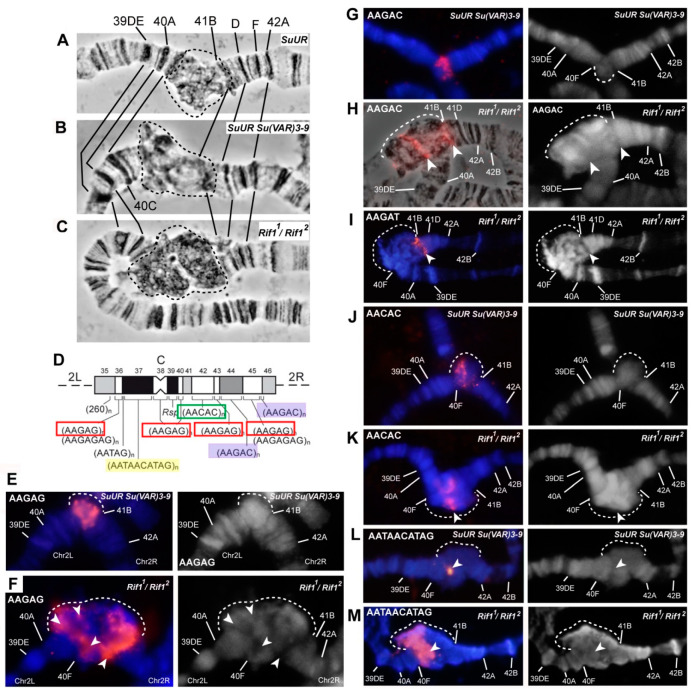
Effect of *Rif1* on the heterochromatin of chromosome 2. (**A**–**C**) Morphology of the pericentromeric regions of chromosome 2 in mutants *SuUR^ES^* (**A**), *SuUR^ES^ Su(var)3-9^06^* (**B**) and *Rif1^1^/Rif2^2^*; (**C**); dotted line indicates the zone of β-heterochromatin morphology. (**D**) A map of the mitotic heterochromatin of chromosome 2 (adapted from [35]). Colors denote the satellite types used for in situ hybridization; (**E**–**M**) in situ hybridization of satellite DNA probes with the polytene chromosome 2 of mutants *SuUR^ES^ Su(var)3-9^06^* (**E**,**G**,**J**,**L**) and *Rif1^1^/Rif2^2^* (**F**,**H**,**I**,**K**,**M**). Probes: AAGAG (**E**,**F**), AAGAC (**G**,**H**), AAGAT (**H**), AACAC (**J**,**K**) and Prodsat (AATAACATAG) (**L**,**M**). White dashed lines indicate the β-heterochromatin of chromosome 2 (the 40F–41B area).

In polytene chromosome 2 of the wild-type larvae, no banding pattern is visible between bands 40A and 41F [4,63]. In *SuUR^ES^* and *SuUR^ES^ Su(var)3-9^06^* mutants, clear-cut bands emerged in the region 41B–F of chromosome 2R [35,63] (Figure 5A,B). In *SuUR^ES^ Su(var)3-9^06^* double mutants, there are additional bands in the 2L chromosome proximal to 40A (Figure 5B). All additional bands arise mainly from the zone of eu–heterochromatin transition and the very distal heterochromatin, and most of the mitotic heterochromatin is composed of structureless β-heterochromatin [35]. In the polytene chromosomes of *Rif1^1^/Rif2^2^* larvae, the regions with a clear-cut banding pattern are very similar to those of *SuUR^ES^* mutants (Figure 5A,C). The β-heterochromatin structure enclosed between bands 40B and 41B looks significantly larger than that in the other lines. To determine which heterochromatin regions are responsible for this increase in heterochromatin mass, we hybridized in situ satellite sequences located in the heterochromatin of chromosome 2. Figure 5D illustrates the positions of the satellite probes used, on a mitotic heterochromatin map of chromosome 2.

A significant part of the heterochromatin of chromosome 2 consists of the alternation of blocks of simple satellites of the AANAN type. The AAGAG satellite is represented by several extended DAPI-negative blocks on chromosome 2R (Figure 5D). On the polytene chromosome 2 of *SuUR^ES^ Su(var)3-9^06^* mutants, it gave sufficiently pronounced signals proximally to 41B and in the region of structureless material (Figure 3D and Figure 5E). In *Rif1^1^/Rif2^2^* mutants, the signals on chromosome 2 are very strong and numerous (Figure 3E, Figure 5F). The most intense signal is located on chromosome 2R proximally to 41B in the region corresponding to the boundary of the β-heterochromatic material. On the mitotic map, the material corresponding to the AAGAG satellite is DAPI negative [38] (Figure 5D). The same is true for polytene chromosomes (Figure 3E and Figure 5F).

The AAGAC satellite is not polytenized in wild-type salivary gland polytene chromosomes [56]. In *SuUR^ES^ Su(var)3-9^06^* mutants, this satellite yielded a pronounced signal, but this signal was usually single and structureless (Figure 5G). In *Rif1^1^/Rif2^2^* mutants, we detected two signals (Figure 5H) probably corresponding to two localization zones of this satellite on metaphase chromosomes: at h44 and at the h45/h46 border (Figure 5D). We can conclude that the material lying between these areas (in particular the AAGAG satellite from h45) is significantly polytenized.

In the polytene chromosome 2 of *SuUR^ES^ Su(var)3-9^06^* mutants, satellite AAGAT showed a clear-cut band-shaped signal proximal to 41B (Figure 3F). In *Rif1^1^/Rif2^2^* mutants, the signal is located at the same position: directly in a DAPI-negative line proximal to 41B (Figure 5I). It can be assumed that the extended block of this satellite is located distally toward the AAGAG satellite block from h45. It is likely that unannotated h59 corresponds to the AAGAT satellite.

The AACAC satellite, specific for chromosome 2, yielded similar signals in the middle of an unstructured heterochromatin block in mutants *SuUR^ES^ Su(var)3-9^06^* and *Rif1^1^/Rif2^2^* (Figure 5J,K). Additional polytenization of this satellite in *SuUR^ES^* mutants has been reported by Makunin et al. [37].

Large DAPI-positive heterochromatin block h37 is formed mainly by the AATAACATAG (Prodsat) satellite. In situ hybridization of the probe specific to this satellite gave a very bright signal covering a large area of the DAPI-positive material (Figure 5M, Figure 3J). This satellite shows the most pronounced difference when compared with the Oregon R and *SuUR^ES^ Su(var)3-9^06^* control lines, which almost do not differ in signal intensity (Figure 3H–J and Figure 5L,M).

Thus, all the satellites forming the blocks of mitotic heterochromatin h44–h46 hybridize with a DAPI-negative band proximal to 41B and the edge of the DAPI-positive block of structureless β-heterochromatin of chromosome 2R. This finding is in good agreement with the data obtained previously on double *SuUR^ES^ Su(var)3-9^06^* mutants [35]. Prodsat together with AAGAG gave the bulk of the big unstructured block in the *Rif1* mutants (Figure 5F,M).

An analysis of the γH2AX distribution suggested that on chromosome 2 in the middle of the β-heterochromatin, a zone effectively recognized by the respective antibodies remains, indicating the presence of underreplicated material (Figure S2). Summing up, on the chromosome 2 of the *Rif1* mutants, satellite sequences are more polytenized than those in *SuUR^ES^ Su(var)3-9^06^* mutants. Nonetheless, this polytenization is not complete; a zone of underreplication remains.

### 3.5. Mutations in the Rif1 Gene lead to the Polytenization of Satellite Sequences Surrounding the Centromere of Chromosome 3

**Figure 6:**
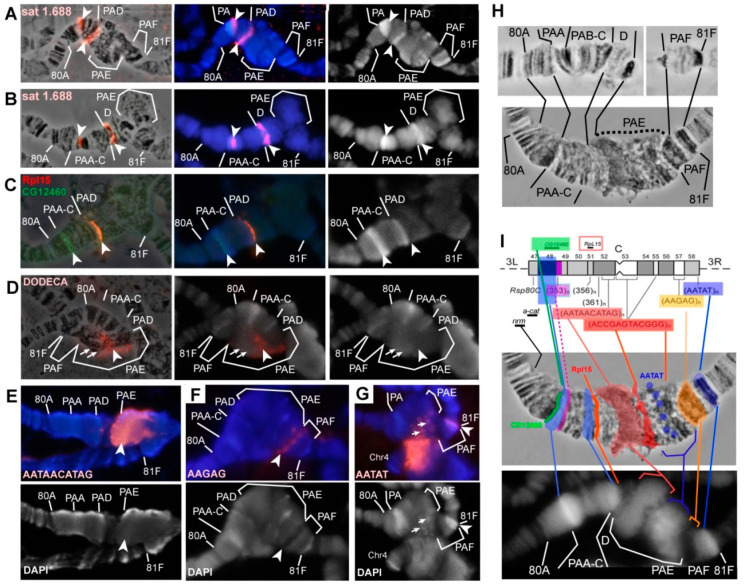
Determining the correspondence between the morphological structures of the pericentromeric heterochromatin of polytene chromosomes in the *Rif1* mutants and the mitotic heterochromatin map of chromosome 3. (**A**,**B**) The probe specific to the het1.688 satellite hybridized with the chromosome of mutants *Rif1^1^/Rif2^2^* (**A**) and *SuUR^ES^ Su(var)3-9^06^* (**B**) (the photo in B is adapted from [60]).(**C**) The probes specific to genes CG12460 (green) and Rpl15 (red) hybridized with the chromosome of *Rif1^1^/Rif2^2^* mutants. (**D**–**G**) FISH of satellite probes with *Rif1^1^/Rif2^2^* polytene chromosome 3; (**D**) Dodeca (ACCGAGTACGGG); (**E**) Prodsat (AATATCATAG); (**F**) AAGAG; (**G**) AATAT. The signals are superimposed with phase contrast images (**A**–**D**, left column) and with DAPI staining (**A**–**D**: middle column, **E**–**G**: upper row). The DAPI staining is presented in black and white. (**H**) Comparison of the morphological structures in Plato Atlantis in mutants *SuUR^ES^ Su(var)3-9^06^* (top) and *Rif1^1^/Rif2^2^* (bottom). (**J**) Correspondence of morphological structures observed in the pericentromeric heterochromatin of chromosome 3 in the *Rif1* mutants to the mitotic heterochromatin map. Different sequences are presented in different colors on the mitotic map and are plotted on the photograph of the polytene chromosome in the corresponding color at a site of their localization.

In wild-type polytene chromosomes, the pericentromeric region of chromosome 3 lying between 8 80C and 81F is composed of poorly structured material, whereas in *SuUR^ES^* mutants and especially in *SuUR^ES^ Su(var)3-9^06^* mutants, extended Plato Atlantis appeared here, characterized by an obvious banding pattern [5,60,63,64]. On the chromosome 3 of *Rif1^1^/Rif2^2^* mutants, we saw structures with the morphology somewhat similar to the Plato Atlantis of *SuUR^ES^ Su(var)3-9^06^* mutants, but it was difficult to identify the correspondence between all the bands on the basis of morphology alone (Figure XH). After DAPI staining, in both cases, two distinct reproducible DAPI-positive bands were seen in Plato Atlantis, which are located in PAA and PAD in double mutants (Figure 6A, left panel). To prove that these bands represent the same material in the polytene chromosomes of both genotypes, we localized the probe specific to satellite 1.688, which was located in DAPI-positive bands in PAA (353-bp satellite) and in PAD (356/361-bp satellite) in double mutants *SuUR^ES^ Su(var)3-9^06^* [60] (Figure 6B). Both DAPI-positive bands in the Plato Atlantis of *Rif1^1^/Rif2^2^* mutants hybridized with the corresponding probe (only the right edge of the band in PAA was found to be stained; Figure 6A). The hybridization signals did not look larger in *Rif1^1^/Rif2^2^* mutants; that is, we did not see a significant effect on the representation of satellite 1.688 on chromosome 3, unlike on chromosome X.

The *CG12460* gene (in the 6th release, it is pseudogene *CR12460*) lies exactly along the left-hand boundary of the DAPI-positive band in PAA in *SuUR^ES^ Su(var)3-9^06^* double mutants, while *Rpl15* is found to the right of the DAPI-positive band in PAD [35,60]. Both probes were localized at the same positions in *Rif1^1^/Rif2^2^* mutants (Figure 6C). Thus, these two DAPI-positive bands can serve as convenient morphological markers of PAA and PAD in both genotypes.

The material that is between these blocks and amounts approximately to 2 Mb [44], which corresponds to approximately two regions (out of 102) on the polytene chromosome map, in double mutants *SuUR^ES^ Su(var)3-9^06^* is represented by PAB–PAC subsections and has a clear-cut banding pattern [60]. In *Rif1^1^/Rif2^2^* mutants, these DAPI-positive blocks were usually found to be closer to each other and an obvious structure was rarely visible between them. Thus, the PAB and PAC sections appear to be “more euchromatic” in *SuUR^ES^ Su(var)3-9^06^* double mutants than in *Rif1* mutants (Figure 6A,B,H).

**Figure 7:**
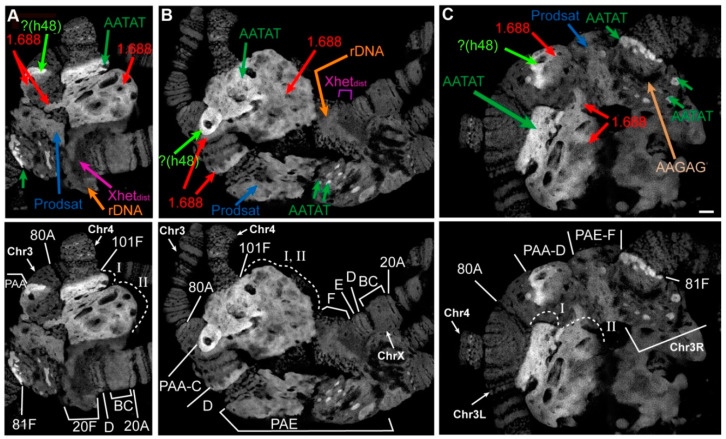
DAPI differentially stains different types of DNA sequences in the heterochromatin of *Rif1* polytene chromosomes. Optical sections (3D-SIM) of the pericentromeric regions of *Rif1^1^/Rif2^2^* polytene chromosomes stained with DAPI. Panels (**A**–**C**) depict three polytene nuclei. The lower row indicates the names of the morphological structures and the upper row shows the correspondence of these zones with different types of DAPI staining to heterochromatin blocks of different DNA composition. Each type of sequence is denoted by a separate color matching among panels (**A**–**C**).

In 3D-SIM–derived optical sections of DAPI-stained polytene chromosomes, it is obvious that the DAPI-positive block in PAA contains a very brightly stained part, which tends to conjugate with the satellite block of AATAT on chromosome 4 (Figure 7). The proximal edge is less brightly stained by DAPI and tends to stick with satellite 359. According to a relatively new updated map of heterochromatin [35], the 353-bp satellite in metaphase chromosomes hybridizes in the proximal part of DAPI-positive block h48. It is likely that this DAPI-positive block h48 corresponds to the DAPI-positive block in PAA in polytene chromosomes. What kind of AT-rich satellite is responsible for the formation of this block remains unknown. Until recently, the 10-bp Prodsat satellite has been erroneously placed in h48 [38], but it was later shown that this satellite is not associated with h48, but rather is located in h52 and is adjacent to the centromere of chromosome 3 on the 3L side [35,46,85].

Another DAPI-positive band reproducible between lines *SuUR^ES^ Su(var)3-9^06^* and *Rif1^1^/Rif2^2^* is located in 81F. This band features a prominent hybridization signal with the AATAT satellite in mutants *SuUR^ES^ Su(var)3-9^06^* and *Rif1^1^/Rif2^2^* (Figure 3A,B and Figure 7C). On mitotic chromosomes, AATAT forms the most distal DAPI-positive block of h58 heterochromatin (Figure 6I).

In *SuUR^ES^ Su(var)3-9^06^* mutants, right and left arms of chromosome 3 are usually separated by a prominent break, in which a cloud of material related to PAE is sometimes visible [60]. This cloud matches the regions of heterochromatin closest to the centromere, in particular, the probe specific to the Dodeca satellite and antibodies against centromeric histone CID bind to this site [60]. A characteristic feature of the Plato Atlantis in the *Rif1* mutants is that the 3L and 3R arms are not separated by a break or thin thread, but rather form a single structure, which is much wider than the main euchromatin chromosome part (Figure 6H). In most cases, the Dodeca satellite gave a signal consisting of many small ones, but sometimes, the signal looked like two relatively structured bands, presumably corresponding to two zones of this satellite on metaphase chromosomes (h53, h55), but sticking together, which is typical for blocks of identical satellites (Figure 6D). Prodsat, flanking the centromere on the left, yielded a very strong signal in the block in the distal part of PAE (Figure 6E). An analysis of chromosome morphology in combination with FISH data tells us that *Rif1* significantly affects the polytenization of these two satellites located on chromosome 3. The material to the right of the zone stained by the Prodsat probe corresponds to the right arm of chromosome 3. After DAPI staining, DAPI-positive granules were visible in this area that hybridize with a probe specific to the AATAT satellite (Figure 6G, arrows, see DAPI-positive points in PAE in Figure 7B,C).

The AAGAG satellite, which forms the DAPI-negative block h57 in mitotic heterochromatin [38], in *Rif1^1^/Rif2^2^* mutants, stained the DAPI-negative zone in PAF (Figure 6F), separated from the DAPI-positive band in 81F by a rather long section, which probably consists of complex DNA: genes and moderate repeats. The *Parp* gene, the *Circea* transposon, and BAC clones enriched in moderate repeats hybridize there [60]. The AAGAG signal is much more intense in the PAF of *Rif1^1^/Rif2^2^* mutants than that of *SuUR^ES^ Su(var)3-9^06^* double mutants (Figure 3D,E), and the block of material in the phase contrast image is much larger too (the black thick most proximal band in PAF in Figure 6H). At the same time, the material between the satellite blocks AATAT and AAGAG in the double mutants has more defined structure: several bands are visible in it (Figure 6H).

In general, a comparison of the morphology and localization of the probes in the heterochromatin of chromosome 3 in mutants *SuUR^ES^ Su(var)3-9^06^* and *Rif1^1^/Rif2^2^* revealed that *SuUR* and *Su(var)3-9* preferentially affect the polytenization of moderately repeated and unique sequences in heterochromatin, but only slightly affect the satellite DNA. *Rif1* affects both the moderate repeats and the satellites. There are areas, such as PAB–C and the decondensed part of PAF, where the influence of *Su(var)3-9* is stronger than that of *Rif1*.

### 3.6. Replication of Pericentromeric Heterochromatin in the Polytene Chromosomes of the Rif1 Mutants

**Figure 8:**
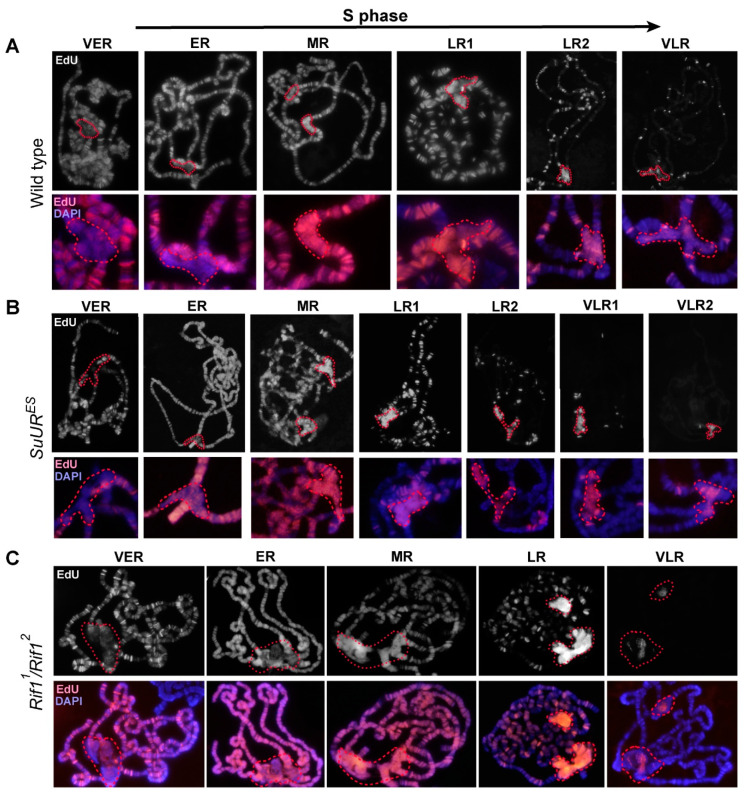
Replication patterns in the salivary glands of the wild-type larvae (**A**), mutants *SuUR^ES^* (**B**) and *Rif1^1^/Rif2^2^* (**C**). Left to right: the stage of early discontinuous labeling (very early replication, VER), the stage of continuous labeling (early replication, ER), the beginning of the stage of late discontinuous labeling (middle S phase, MR), the stage of discrete labeling (late S phase, LR) and the very late S phase (VLR). The stage classification is borrowed from ref [32]. Chromocenters are surrounded by dashed lines.

It is known that the Rif1 protein is involved in the process of late replication of satellite sequences during embryogenesis [31]. Therefore, one would expect that in the *Rif1* mutants, the satellite DNA would replicate much earlier than normal, and this is what leads to additional polytenization of the satellite sequences. To verify this assumption, we examined replication timing in the polytene chromosomes of salivary glands of the *Rif1* mutants and compared it with the timing in the control lines. Figure 8 presents a set of successive patterns of EdU incorporation into polytene chromosomes; these patterns reflect the consequent stages of the S phase in Oregon R and mutants *SuUR^ES^* and *Rif1^1^/Rif2^2^*. In general, in all the three lines, replication patterns match those described for the polytene chromosomes of wild-type larvae in numerous papers published in the 60 s through 80 s (see references in [4,17]) and are easily categorized according to the standard classification: early discontinuous labeling (several sites in decondensed chromatin are labeled), continuous labeling (almost all structures visible in polytene chromosomes are labeled), the beginning of the stage of late discontinuous (discrete) labeling (the chromocenter and most of the bands are labeled), the late S phase (the label is detectable in the chromocenter and at discrete sites corresponding to large bands), the very late S phase (labeling exclusively in pericentromeric heterochromatin; this stage is absent in wild-type larvae, but is present in *SuUR^ES^* mutants).

At the earliest stage of the S phase, i.e., the stage of early discontinuous labeling in all three lines (Figure 8A–C, VER), heterochromatin was almost unlabeled. At the stage of continuous labeling, when EdU was almost totally detectable throughout the polytene chromosome arms (Figure 8A–C, ER), there was some EdU signal in pericentromeric heterochromatin, but the signal was weaker than that the chromosome arms. Thus, a mutation in the *Rif1* gene does not lead to massive early replication of pericentromeric heterochromatin. At the stage of late discrete labeling, when the signals along the chromosome arms were visible exclusively in the discrete bands, the signal in the chromocenter intensified (Figure 8A–C, MR). In the control lines, the signal intensity in the chromocenter was comparable to that in the chromosome arms (Figure 8A,B, MR), and in the *Rif1* mutants, it was brighter than all other signals in the nucleus (Figure 8C, MR). At later stages, when the signals in the arms were concentrated mainly in intercalary heterochromatin regions (Figure 8C, LR), the signal in the chromocenter of the *Rif1* mutants became so intense that it was impossible to take pictures with the same exposure settings for the bands and for the chromocenter without overexposure of the chromocenter. This signal pattern is unique to the *Rif1* mutants and has never been described for wild-type larvae and *SuUR^ES^* mutants. Stages of very late replication, where the signal was detectable only in the chromocenter, were documented too (Figure 8C, VLR). Such stages are not characteristic of wild-type polytene chromosomes (Figure 8A, VLR), but are described for *SuUR* mutants (Figure 8B, VLR1,2) [29,86].

It should be noted that we were unable to obtain the pattern where only several intercalary heterochromatin bands per nucleus are replicated in addition to the chromocenter (Figure 8C, LR-VLR). This pattern is a feature of *SuUR^ES^* mutants (Figure 8B, LR2–VLR1) [86]. Instead, we saw nuclei in which quite a lot of late-replicating bands were labeled, but the nuclei differed in brightness of the labeling signals from bright enough to very weak, almost invisible against the background of a bright signal in the chromocenter (data not shown). This result implies a specific (different from *SuUR^ES^* effects) influence of the *Rif1* mutations on the course of replication not only in the pericentromeric heterochromatin, but also in the euchromatin arms. In this study, we did not analyze this phenomenon in detail and instead focused on heterochromatin regions.

**Figure 9:**
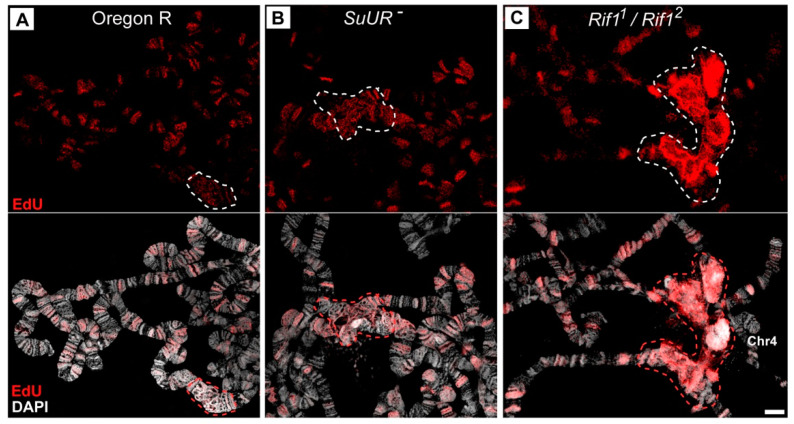
Analysis of the replication patterns corresponding to late discrete labeling using super-resolution 3D-SIM of the polytene chromosomes of wild-type larvae and the *SuUR* and *Rif1* mutants. Visual fields inside salivary gland nuclei from the Oregon R (**A**), *SuUR^ES^* (**B**) and *Rif1^1^/Rif2^2^* (**C**) larvae at the late discrete labeling replication stage. Dashed lines indicate the chromocenters. Scale bar 5 µm.

We compared the replication patterns at the late discrete labeling stage between the wild type (Figure 9A) and mutants *SuUR^ES^* (Figure 9B) and *Rif1^1^/Rif2^2^* (Figure 9C) by super-resolution 3D-SIM microscopy. An analysis of the distribution of signals of short-term (7 min) EdU incorporation revealed that in the wild-type chromosomes and in *SuUR^ES^* mutants, the overall level and appearance of the signals in the pericentromeric regions (circled by dotted lines) did not significantly differ from the signals in the late-replicating bands. In the *Rif1* mutants, the distribution of EdU signals in the chromocenter was significantly different. The total signal was very intense, the individual dotted signals merged into a solid color (compare to discrete point signals in panels A and B). Because EdU incorporation, photo shooting and photo processing were carried out under similar conditions for panels A–C, we can conclude that at this stage of the S phase, significantly more DNA is produced during the EdU incorporation period in the *Rif1* mutants than in wild-type and *SuUR^ES^* chromosomes.

**Figure 10:**
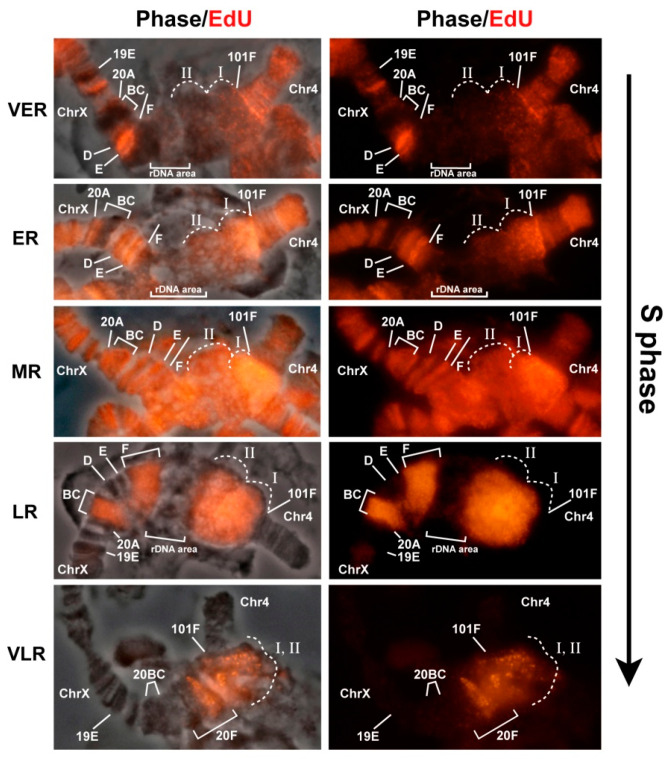
Sequential patterns of pulse EdU incorporation into the pericentromeric region of the X-4 chromosome. The left panel is a superposition of the EdU signal on a phase contrast image, and the right panel depicts EdU data. The stages of replication are presented in accordance with Figure 8. The exposure parameters are not identical among different stages and were selected for the most convenient mapping of pericentromeric signals.

Figure 10 illustrates the sequential replication patterns in joined chromosomes X and 4. At the VER stage (Figure 10), separate sites of intense EdU incorporation were visible in the euchromatic arms of polytene chromosomes, followed by the stage of ER, when labeling was almost continuous in the arms. During these stages in the pericentromeric region of chromosome X, signals were absent in the zone of large block 20BC, which is likely an islet of heterochromatin inside euchromatin [35,62], and in region 20F, corresponding to the distal heterochromatin of chromosome X [35,58,62]. The euchromatin region in 20D–E was found to be actively labeled with EdU. The area of the nucleolar organizer did not incorporate EdU at this stage. That is, in general, we saw continuous labeling in euchromatin and a complete absence of labeling in heterochromatin. The heterochromatin zone adjoining the euchromatin part of chromosome 4 violated the general pattern. There was a very bright band of the signal in the 101F region, behind which lied a clearly visible cloud of point signals that became very bright at the ER stage in the zone of the localization of the AATAT satellite. At the MR stage, EdU was detectable in all compact bands of the chromosome arms and throughout the heterochromatin, while the block corresponding to the AATAT satellite at the base of chromosome 4 featured a very bright and solid EdU signal. The very high brightness of the signal in the region of the AATAT satellite can be attributed to the stoichiometric incorporation of EdU, which is an analog of thymidine, and the signal intensity should be proportional to the AT percentage, which is maximal (100%) in the AATAT satellite. This explanation is supported by quantification of fluorescence intensity values in the ImageJ software (https://imagej.nih.gov/) along the heterochromatin at this stage (Figure S3). The greatest difference in fluorescence intensity was 30%–40%, which matches the difference in the AT composition between different zones of heterochromatin (57%–100% according to ref. [38]). At the LR stage, labeling remained only in late-replicating bands, including 19E, 20BC and 20F. The base of chromosome 4, both the AATAT satellite unit (zone I) and the proximal heterochromatin of chromosome X (zone II), manifested strong EdU incorporation (Figure 10, stage LR). The heterochromatin signals were so strong that the signal in 19E was almost invisible when exposure was set for typical heterochromatin areas. The last regions to be replicated were individual zones at the base of chromosome 4, according to localization possibly representing islands of other satellites, such as AAGAG or AAGAT in satellite block AATAT (zone I) and a part of the zone corresponding to satellite 1.688 (zone II). Thus, in the X-4 chromosomes, the heterochromatin regions enter replication rather late, but the AATAT satellite zone stands out: it enters replication earlier than other heterochromatin regions do, with a gradual increase in the replication signal, indicating the emergence of new active replicons during the replication.

**Figure 11:**
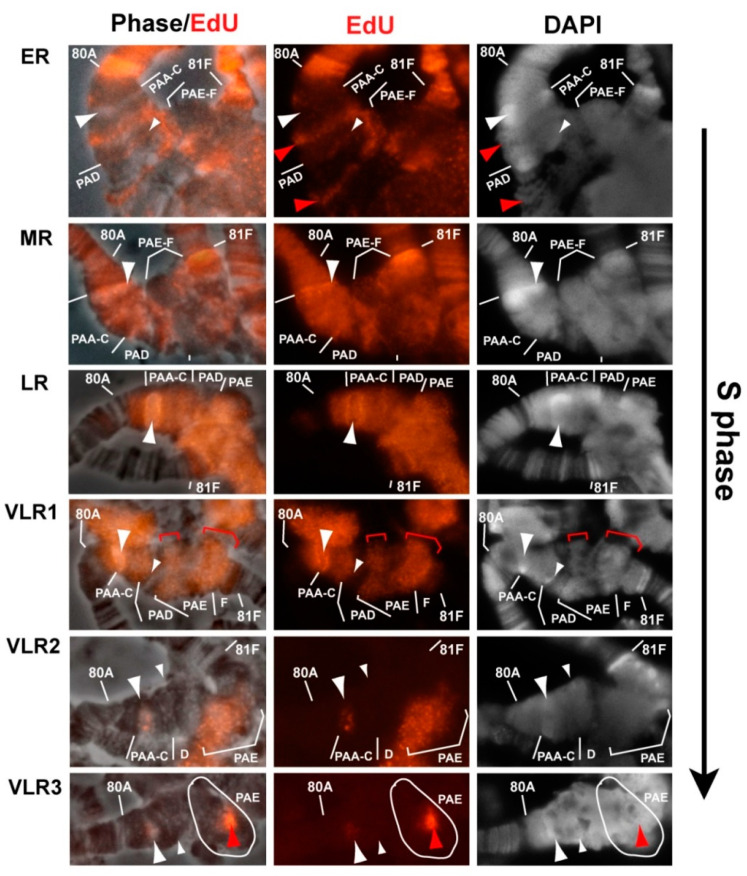
Sequential patterns of pulse EdU incorporation into the pericentromeric region of chromosome 3. The left panel is a superposition of the EdU signal on a phase contrast image, the middle panel depicts EdU data, and the right panel illustrates DAPI staining. The red arrowheads and brackets mark the positions of EdU signals. A white outline indicates the PAE area. The stages of replication are given in accordance with Figure 8, but the VLR stage is divided into three: VLR1–3. The exposure parameters are not identical among different stages and were selected for the most convenient mapping of pericentromeric signals.

In polytene chromosome 3, even in the very early stages of the S phase (VER), we noticed obvious replication signals within Plato Atlantis (Figure 11, VER). The simultaneous localization of EdU and antibodies to the active form of RNA polymerase 2 showed that all regions of VER tended to be approximately in or near the localization areas of the active form of RNA polymerase II (RNAse polymerase II phosphorylated on serine 5, RNAPII Ser5P; Figure S4, VER). In general, however, with the exception of these sites, the region from 80A to 81F was devoid of transcription and replication signals at the VER stage. At the stage of continuous labeling (stage ER), most of the pericentromeric region of chromosome 3 from 80A to 81F showed a continuous signal comparable in intensity with the signal in the chromosome arms (Figure 11, ER). The gaps were visible in both DAPI-positive areas in PAA and PAD (marked with an arrowhead and arrow). The zone corresponding to the centromeric satellites—the Prodsat satellite and the right arm heterochromatin, which looks like a huge chromatin block without a banding pattern—contained an uneven weak signal, with zones of dips, in particular, a large dip was visible in the center of this area. A comparison with the distribution of RNAPII Ser5P revealed that both transcriptionally active regions of the pericentromeric heterochromatin and individual zones where the transcriptional signal is not expressed are replicated. The later stage (MR) featured the appearance of intense EdU signals in the DAPI-positive zones in PAA and 81F, which became even more contrastingly bright compared to other regions of chromosome 3. Meanwhile, the PAE-PAF zone still gave a weak replication signal (Figure 11 MR). At the LR stage, the entire pericentromeric region incorporated EdU relatively evenly (and very brightly, see above). Upon transition from the LR stage to VLR (characterized by the absence of signals in the chromosome arms), gaps emerged in the continuous labeling of heterochromatin. The first gaps exactly matched the zones of active transcription (Figure S4, LR). Then, other regions of heterochromatin exited replication, with the DAPI-positive block in the PAA and the area of pericentromeric satellites (Prodsat and Dodeca) in PAE being the last to exit (Figure 11, VLR1–3).

Therefore, the analysis of the replication schedule in chromosome 3 indicates that the zone of pericentromeric satellites in PAE, which is most different between the *Rif1* and *SuUR^ES^ Su(var)3-9^06^* mutants, shows a weak EdU signal until the late stages of the S phase, but at stages LR and VLR, features very intense labeling. These data suggest that in the late S phase, multiple replication initiation events take place there.

Given that the late replication pattern in chromosome 3 of *SuUR^ES^ Su(var)3-9^06^* mutants has not been previously published, we analyzed the replication in these mutants. Figure S5A shows that the overall level of the replication signals in the pericentromeric heterochromatin at the LR stage was comparable to that in the late-replicating bands. Bright signals were present in DAPI-positive AT-rich zones in PAA and 101F. By the time chromosome arms had already completed replication, almost all the heterochromatin was labeled, with several gaps (Figure S5B). After analyzing many nuclei, we can say that the sites where replication is visible the latest are DAPI-positive bands in PAA (Figure S5C,D) and the base of chromosome 4 (101F). The diffuse signal of very late replication was also visible in PAE (Figure S5C). This zone is significantly underreplicated in *SuUR^ES^ Su(var)3-9^06^*, which is why the signal could be so weak. In general, the order of completion of replication in the pericentromeric region of chromosome 3 was surprisingly similar between the Rif1 mutants and *SuUR^ES^ Su(var)3-9^06^* double mutants.

**Figure 12:**
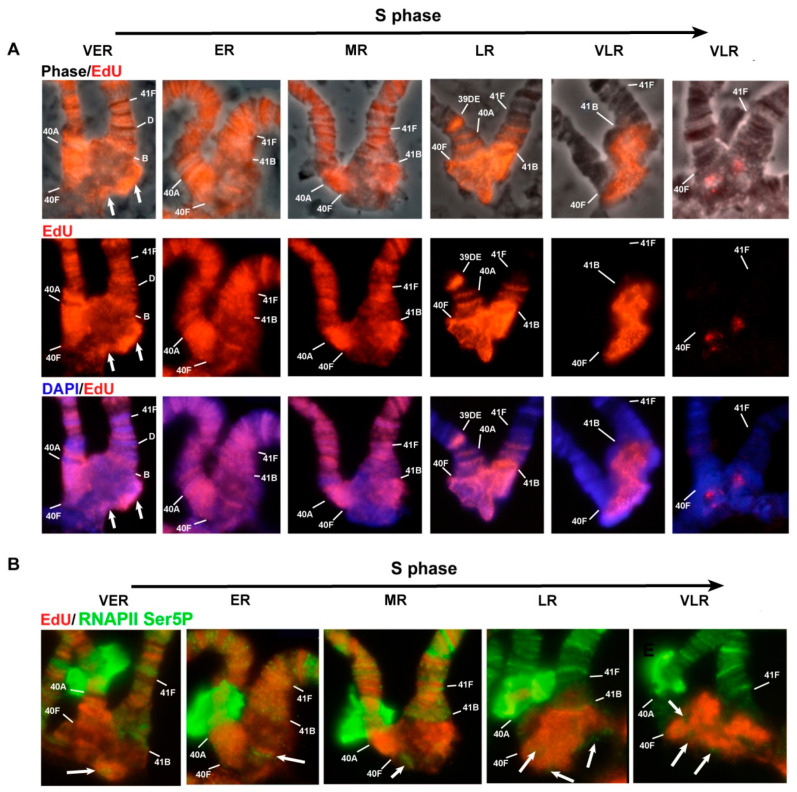
Replication patterns in the pericentromeric region of chromosome 2 and their relationship with the distribution of active transcription. (**A**) EdU incorporation into polytene chromosomes of *Rif1^1^/Rif2^2^* mutants at successive stages of the S phase. The upper row: a superposition of EdU and a phase contrast image, the middle row: EdU data and the bottom row: EdU (red) and DAPI (blue). (**B**) Simultaneous detection of EdU (red) and antibodies to RNAPII Ser5P (green).

On chromosome 2, a substantial part of heterochromatin does not have a pronounced reproducible structure in the *Rif1* mutants. A detailed analysis of replication patterns (Figure 12) showed that at the early stages of the S phase (VER and ER), pericentromeric zones 2L (40A–40F) and 2R (41B–F), in which there was a clear banding pattern, were actively replicated. At this time point, the bulk of heterochromatin contained individual EdU incorporation spots, which were often localized on the surface of the structure (arrows). In a comparison with the distribution of RNAPII Ser5P (Figure 12, stages VER and ER), it was evident that these signals tended to be in or near the zones where transcription was observed (arrows). At the MR stage, a zone of active replication appeared in heterochromatin 2R, while the regions surrounding transcriptionally active chromatin in the center of the heterochromatin and in region 41 (Figure 12B, stage MR, arrow) were no longer replicated. At the LR stage, the entire heterochromatin block in 2R turned out to be intensively labeled, but the heterochromatin spots associated with active transcription also tended to be devoid of labeling (Figure 12B, LR and VLR stages, arrows). At later stages (VLR), the EdU signal was observed in the central region of the heterochromatin block, gradually “contracting” to DAPI-negative spots, suggesting that the DAPI-negative AAGAG satellite completes replication very late.

In general, on chromosome 2, the chromatin that formed the central block tended to contain a portion of early replicating chromatin on the surface of the structure, whereas in the course of the S phase, replication moved from the surface to the central zone. Actively transcribed chromatin was likely to be located on the surface.

Overall, a detailed analysis of replication patterns in individual regions of heterochromatin revealed that in the *Rif1* mutants, the transcriptionally active regions of heterochromatin are replicated very early, whereas the bulk of heterochromatin enters replication at the LR stage. Zone I (at the base of chromosome 4), which hybridizes with the AATAT satellite, stands out among other areas and begins to incorporate EdU at the early stages of replication and shows a gradual expansion and increase in signal brightness.

## 4. Discussion

Underreplication in polytene chromosomes is a common phenomenon seen in a wide range of organisms [87]. In *Drosophila*, underreplication is characteristic of nearly all studied tissues where polytene chromosomes appear [8,87,88,89]. Differential underreplication in these tissues suggests that underreplication is a regulated process [28,89,90]. It is believed that underreplication can serve as an additional mechanism for the silencing of heterochromatin sequences and tissue-specific genes. In addition, underreplication represents existing saving of cell resources [6,26,87,91,92]. On the other hand, underreplication can also be considered a passive consequence of features of the endocycle, which makes underreplication possible in principle. Constant conjugation of sister chromatids preserves chromosome integrity even under the conditions of underreplication of extended chromosome sections. The intra-S-phase checkpoint and apoptosis are suppressed in endocycling cells [10,18,19,20,93], thereby allowing the cell to continue the endocycle in the presence of extended underreplicated areas. The notion that underreplication is more likely a passive consequence of the endocycle is supported by the fact that mutations that significantly suppress underreplication in the polytene chromosomes of *D. melanogaster* have little or no effect on larval development time and viability [5,27,31]. Even though there is no unambiguous answer to the question of the functional significance of underreplication and its differential regulation in different tissues, mutations that suppress underreplication in *Drosophila* pericentromeric heterochromatin are of great practical importance because they allow for the use of polytene chromosomes for studies on heterochromatin organization.

The repetitiveness of heterochromatin sequences is a challenge for researchers. Despite the rapid development of molecular and bioinformatic methods for analysis and assembly of large sections of heterochromatin in recent years [41,42,43,44,45,46,47], cytological approaches remain an indispensable tool for verification of the assembly and the analysis of mutual arrangement of heterochromatin contigs [35]. In salivary gland polytene chromosomes of wild-type larvae, heterochromatin sequences are strongly underrepresented. Although unique sequences and some moderate repeats are present there at a proportion of up to 100%, these sequences alternate with completely underreplicated ones and the resulting network structure does not allow for visualization of individual sequences [54,55].

Several *D. melanogaster* genotypes have been found in which additional polytenization of the heterochromatin regions takes place. The *otu^11^* mutation leads to the formation of classic polytene chromosomes in nurse ovarian cells. In these cells, underreplication begin not with the first, but with the fifth replication cycle; therefore, heterochromatin sequences are more polytenized [94]. This phenotype has been used for cytogenetic analysis [57,59]. The most useful for cytogenetic studies is the combination of mutations *SuUR^ES^* and *Su(var)3-9^06^*, which have made a big contribution to the assembly of the heterochromatin part of the *D. melanogaster* genome, 6th release [35]. In the double mutant, there is significant polytenization of heterochromatin blocks enriched in moderately repeated DNA and unique sequences. As a result, the distal heterochromatin of chromosome X, the zones of eu–heterochromatin transition of chromosome arms 2R and 3L, and a major part of the heterochromatin of chromosome 3L adopt a defined structure. Nevertheless, the chromosome X proximal heterochromatin adjacent to the centromere region of chromosome 3 and the entire Y chromosome in these mutants remain completely non-polytenized. Overall, it has been shown that the effect of these mutations on satellite DNA, which constitutes ~21% of the *Drosophila* genome [36,38], is very small [35]. A significant limitation of the *SuUR^ES^ Su(var)3-9^06^* model is that the Su(VAR)3-9 protein is one of the key players in heterochromatin formation [95,96] and the regions polytenized in response to the mutation do not have typical protein composition, which makes it impossible to study the epigenetic properties of heterochromatin in such a model.

In this study, we demonstrated that *Rif1* mutations lead both to polytenization of most of the regions polytenized in the *SuUR^ES^ Su(var)3-9^06^* line and the regions for which polytenization has never been described before. We detected polytenized satellites that formed large blocks, in particular, these are large blocks of simple satellites AATAT and AAGAG, satellite 1.688 from the proximal heterochromatin of the Chromosome X, satellites AATAACATAG (Prodsat) and ACCGAGTACGGG (Dodeca) located around the centromere of chromosome 3. In addition, for the first time, we visualized in polytene chromosomes a structure that we consider to be chromosome Y. Munden et al. [27] have suggested that in *Rif1* mutants, underreplication is fully reversed. According to the size of the heterochromatin blocks, we believe that polytenization is not complete. For example, chromosome Y in terms of DNA is comparable to the whole chromosome X. In addition, we showed that γH2AX sites continue to be detectable in the chromocenter of the *Rif1* mutants; γH2AX in *Drosophila* polytene chromosomes correlates clearly with sites of underreplication [21].

All the zones of *Rif1*-specific polytenization match the largest blocks of satellite DNA from *Drosophila* pericentromeric heterochromatin. Thus, the satellite block 1.688 from chromosome X contains 11 Mb of DNA [38] and forms the largest newly polytenized structure in a polytene chromosome of the *Rif1* mutants. The related satellite blocks in chromosome 3 contain significantly less DNA [38,44,83], and the morphology of these blocks does not differ between the *Rif1* mutants and *SuUR^ES^ Su(var)3-9^06^* mutants. AATAT in chromosome 4 occupies 2.7 Mb of DNA, and again we noted a significant increase of the corresponding polytene chromosome structure in the *Rif1* mutants. The same was true for AAGAG satellite blocks (on the X chromosome: 1.2 Mb, on the 4th chromosome: 170 kb, in chromosome 2: 5.5 Mb, and in chromosome 3: 1 Mb [38]). We saw a noticeable increase in signal on chromosomes 2 and 3 in the *Rif1* mutants, but on chromosome 2, it was more pronounced. On chromosome 3, the block associated with the Prodsat satellite (1.6 Mb) is significantly larger, but not the block associated with the Dodeca satellite, composed of two significantly smaller blocks [85]. The rRNA gene cluster on the X chromosome is almost 3 Mb [38]. Normally, only 4% is polytenized [5], indicating the inactivation of the internal origins of replication. In the *Rif1* mutants, rDNA has significantly greater representation.

In their study [61], He et al. created a set of heterochromatin-specific probes, which were used for comparative genomic hybridization analyses. Those authors demonstrated that there is a correlation between the repetitiveness of the probes and the degree of their underreplication in salivary gland cells (r = −0.80). This phenomenon can be explained by the following scenario: extended satellite paths do not initiate replication in wild-type polytene chromosomes, whereas moderate repeats flanking or embedded in these paths do initiate replication. Research on the effects of SUUR on intercalary heterochromatin and amplification of the chorionic gene cluster has shown that SUUR can act on replication forks and *SuUR* mutation is sufficient for complete polytenization of intercalary heterochromatin regions as large as hundreds of kilobase pairs without additional activation of internal origins. It can be assumed that in the satellite blocks not exceeding several hundred kilobase pairs, in the presence of mutation *SuUR^ES^*, additional polytenization occurs owing to the replication forks coming from complex DNA islands. The longer blocks cannot be polytenized without additional activation of internal origins, and it is precisely this type of activation that is observed in the *Rif1* mutants. According to polytene chromosome morphology, almost all regions polytenized in *SuUR^ES^* mutants are also polytenized in the *Rif1* mutants. We assume that in pericentromeric heterochromatin, Rif1 has two mechanisms of action: a SUUR-dependent influence on replication fork progression and a SUUR-independent effect on origin activation.

Of note, in heterozygotes, we saw an intermediate effect, which is consistent with the data of Armstrong et al. [65] about the impact of heterozygous *Rif1* mutations on replication timing. The similarity of the heterozygous *Rif1* phenotype and the homozygous SuUR phenotype suggests that SUUR-dependent effects require less Rif1 protein, whereas the SUUR-independent effect has a higher threshold of Rif1 concentration.

Su(VAR)3-9 is another regulator of replication timing in heterochromatin, although Su(VAR)3-9 affects only a small fraction of heterochromatin [62,97]. A comparison of polytene chromosome regions of additional polytenization between the *Rif1* mutants and *SuUR^ES^ Su(var)3-9^06^* double mutants indicates that despite the general similarity, some zones are under a specific influence of the Su(VAR)3-9 protein. These zones include chromosome X distal heterochromatin, which forms a giant pseudo-puff on the *Su(var)3-9^06^* background [62]. It has been reported earlier that in the presence of double mutation *SuUR^ES^ Su(var)3-9^06^*, the distal heterochromatin of chromosome X replicates much earlier than it does in the *SuUR^ES^* mutant [62], and we can assume the specific influence of Su(VAR)3-9 on the replication initiation.

In *Drosophila*, the euchromatin part of the genome is relatively uniform in AT/GC composition; therefore, staining with intercalating dyes, in particular, DAPI, gives uniform brightness to metaphase chromosomes and reflects the amount of DNA and compactness in polytene chromosomes (reproduces the banding pattern seen in phase contrast images). Mitotic heterochromatin is characterized by alternating regions of different intensities after staining with intercalating dyes. There is a correlation between the brightness intensity and AT content [38,98]. The AATAT satellite binds intercalators very effectively, whereas AAGAG does not. As shown recently, the inability of AAGAG to bind intercalating substances is related to its propensity to form noncanonical DNA structures, i.e., triplex DNA [99]. This differential staining has proven to be convenient for building mitotic heterochromatin maps: heterochromatin has been subdivided into 61 regions with diverse cytological features, designated h1 to h61 [50,100]. Given that satellite DNA is not polytenized in wild-type polytene chromosomes and almost nonpolytenized in mutants *SuUR^ES^* and *SuUR^ES^ Su(var)3-9^06^*, DAPI-negative and DAPI-positive zones have never been highlighted, excepttwo DAPI-positive zones in Plato Atlantis (heterochromatin of chromosome 3), corresponding to the satellites from group 1.688 [21,60]. In our study, in the *Rif1* mutants, the additional polytenization of large blocks of different types of satellites gave rise to pronounced alternation of DAPI-negative and DAPI-positive zones in heterochromatin (Figure 7). Thus, according to the DAPI staining, the AATAT satellite, the 1.688 satellite blocks in chromosome 3, and the AAGAG satellite in chromosomes 2 and 3 are clearly visualized. Prodsat stands out as a separate structure. The boundary between the hone of rDNA and satellite 1.688 of chromosome X is clearly visible. This “differential staining,” which reveals the blocks of different satellites, allows us to study the features of chromatin organization associated with different satellites. Accordingly, we noticed a tendency for the regions with similar DNA composition to fuse. For DAPI-positive areas, we documented a tendency to form round drop structures. It is known that the AATAT satellite has unique chromatin composition, in particular, it is significantly depleted of histones [42]. Jagannathan et al. [101] have shown that the AATAT satellite in chromosomes X, Y and 4 and the AATAACATAG satellite (Prodsat) in autosomes play a key role in the formation of the chromocenter. They bind to the D1 and Prod proteins, respectively, while the concentration of these proteins on DNA is very high and, according to those authors, the behavior manifested by these satellite DNA–binding proteins may reflect liquid-droplet–like properties of heterochromatin [102,103]. The phenomenon of liquid–liquid phase separation of different types of chromatin is based on the ability of proteins possessing intrinsically disordered regions (for example, protein HP1 and histones) to assemble on a DNA matrix at a very high local concentration, which leads to their release into a separate liquid phase. Such phase condensates tend to merge if they have similar protein composition and are likely to have a round shape [104,105]. The same properties characterize the blocks of satellite DNA polytenized in the *Rif1* mutants. Therefore, satellite DNA regions in polytene chromosomes of the *Rif1* mutants may become unique tools for studying the phase separation of different types of heterochromatin.

Because the *Rif1* mutants, just as *SuUR^ES^ Su(var)3-9^06^* double mutants, possess polytenized and structured heterochromatin, we can investigate replication timing of the heterochromatin regions by comparing replication patterns at different stages of the S phase. Our assay of EdU pulse incorporation suggests that in polytene chromosomes of the *Rif1* mutants, generally, the change of replication patterns follows a scenario similar to that in wild-type and *SuUR^ES^ Su(var)3-9^06^* lines: a significant part of heterochromatin is replicated mainly at the end of the S phase when most of euchromatin has already finished replication. This notion implies that the mutation in the *Rif1* gene did not cause a global change in the timetable of replication for different genomic regions. This observation is in good agreement with the data of Armstrong et al. [65] on ovarian follicular cells and larval imaginal disc cells. At the same time, there is a striking difference in the intensity of replication signals during the late S phase, and we distinguished successive stages in which gradual amplification and then attenuation of the signal was observed. This finding indirectly indicates the involvement of an increasing number of replicons during the late S phase.

In our work, all the effects observed in *Rif1* null mutations were also detected in the *Drosophila* line with the mutation in the PP1 phosphatase–binding domain. This result suggests that all the observed phenomena are associated with the influence of Rif1 on dephosphorylation of MCM, which may affect both the timing and probability of activation of replication origins as well as the movement of replication forks. Studies on the mechanism of Rif1 action have led to the notion that the binding of Rif1 to a late origin and its presence increases the threshold kinase concentration necessary for activation of MCM [31,106,107]. Therefore, wild-type Rif1 lowers the number of potential origins and replication efficiency.

Our data indicate that, normally, Rif1 blocks the activation of all replication origins located in satellite DNA blocks in polytene chromosomes. In the absence of Rif1, satellite origins can activate replication, but enter replication after the bulk of euchromatin origin has activated replication. It can be theorized that heterochromatin origins have a lower activation threshold even in the presence of a Rif1 mutation, and in the competition for the limiting factors necessary for replication initiation, they lose to early origins and are activated at the end of the S phase, when most of euchromatin has completed replication.

## 5. Conclusions

Morphological analysis of pericentromeric regions and the labeling patterns of in situ hybridization of heterochromatin-specific DNA probes allowed us to come closer to elucidating the mechanism of action of a conservative regulator of replication timing, the Rif1 protein, in the heterochromatin of *D. melanogaster* polytene chromosomes. It was found that the new morphological structures in the Rif1 polytene chromosomes are produced by different DNA regions, in particular, by satellites. Moreover, different satellite blocks can be easily differentiated by the intensity of DAPI staining, which is a unique tool for further research into chromatin composition of satellite blocks in *Drosophila* heterochromatin. It was demonstrated that in the Rif1 mutants, there is additional polytenization of the largest blocks of satellite DNA as well as partial polytenization of chromosome Y, which has never been visualized in the polytene chromosomes of *Drosophila*. Detailed analysis of replication patterns showed that in the Rif1 mutants, similarly to the wild type, most of the heterochromatin gets replicated during the late S phase, but a significant increase in the number of heterochromatin replicons occurs. These results suggest that Rif1 regulates the activation probability of heterochromatic origins in the satellite DNA region rather than the time of origins’ activation.

## Figures and Tables

**Table 1:** Description of the mutations used in this work.

Genotype	Allele				Reference
	Allele Name	Chromo- some	Mutation Description	Effect on Viability and Fertility	
***Rif1* mutants**					
*w^118^*; *Rif1^1^*	*Rif1^1^*	2	Deletion obtained via CRISPR-based mutagenesis; frame shift mutations at amino acid position 14. No detectable Rif1 protein. FBal0343570	Viable and fertile. A modest defect in the embryonic hatch rate relative to wild-type flies.	[27]
*w^118^*; *Rif1^2^*	*Rif1^2^*	2	Deletion obtained via CRISPR-based mutagenesis. Frame shift mutations at amino acid position 11. No detectable Rif1 protein. FBal0343571		[27]
*w^118^*; *Rif1^1^/Rif1^2^*	*Rif1^1^/Rif1^2^*	2	The heterozygous combination used in [27] as a *Rif1*-null mutant.		[27]
*w^118^*; *Rif1^PP1^*	*Rif1^PP1^*	2	A *Rif1* allele with a mutation in the PP1-interacting motif. FBal0343572	Low viability and sterility of homozygotes, presumably owing to the off-target effect.	[27]
*w^118^*; *Rif1^KO^*	*Rif1^KO^*	2	A *Rif1^KO^*-null allele obtained by replacing the *Rif1* open reading frame with a visible 3xP3-DsRed marker via CRISPR-based mutagenesis. FBal0344229	A reduction in survival, a reduced male-to-female ratio. Embryos laid by *rif1* mutant mothers have a reduced hatch rate.	[31]
**Other**					
*In(1)w^m4h^*; *Rif1^1^/Rif1^2^*	*In(1)w^m4h^*	*1*	Inversion *In(1)w^m4h^* having break points 3C1-2 and 20F (h28). FBab0004257		
*SuUR^ES^ Su(var)3-9^06^*	*SuUR^ES^*	3	FBal0091620	Normal with respect to morphology, viability and fertility. C-heterochromatin staining does not reveal abnormalities in the karyotype [5].	[60,62]
	*Su(var)3-9^06^*	3	FBal0016562	The *SuUR^ES^ Su(var)3-9^06^* line is viable and fertile, but eventually loses its viability and the line is maintained in a laboratory fund in a heterozygous state.	

## Supplementary Materials

The following are available online at https://www.mdpi.com/2073-4409/9/6/1501/s1, **Table S1**. The list of DNA probes used for FISH. The method and parameters of their preparation and labeling are indicated. N, M and L: variable parameters in the PCR without a template program. N and M are increasing annealing temperatures and L is the extension temperature. **Table S2.** The read alignments number of the sequenced microdissection library against the database of repeated elements RepBase. **Figure S1.** Effects of *SuUR* and *Rif1* on the nucleolus. In situ hybridization of the 28S rDNA probe on polytene chromosomes of Oregon R (A), *SuUR^ES^* (B) and *Rif1^1^/Rif1^2^* (C) larvae. Dotted lines approximately outline the areas of nucleoli. **Figure S2.** In the chromocenter of the *Rif1* mutants, underreplication zones remain. Localization of anti-γH2AX antibodies in the pericentromeric regions of joint chromosome X–4 (A), chromosome 2 (B) and all five chromosome arms (C) in *Rif1^2^* mutants. **Figure S3.** Quantification of fluorescence intensity after EdU detection along the heterochromatin at the MR–LR stage. The fluorescence intensity was determined along the yellow line passing through the bright heterochromatin block at the base of chromosome 4. **Figure S4.** Interrelation of replication patterns in the pericentromeric region of chromosome 3 with the distribution of active transcription in polytene chromosomes of *Rif1* mutants. The left column: the VER stage of the S phase, the middle column: ER and the right column: the LR stage. The figure illustrates the distribution of RNAPII Ser5P (first row), EdU (second row), an overlay of red and green with a phase contrast image (3rd row) and DAPI staining (4th row). Dotted lines along the chromosome mark the areas where the signal was detected. **Figure S5.** Late replication stages in the chromosomes of *SuUR^ES^ Su(var)3-9^06^* mutants; (A) General view of the EdU incorporation pattern at the stage of LR. B–D. Three subsequent stages of very late replication.
