# Supplementary material for: Effects of Mutations in the Drosophila melanogaster Rif1 Gene on the Replication and Underreplication of Pericentromeric Heterochromatin in Salivary Gland Polytene Chromosomes
Source: Cells. 2020 Jun 19;9(6):1501. doi: 10.3390/cells9061501 (PMC7349278; doi:10.3390/cells9061501)
Supplement: Supplementary file 1 [file cells-09-01501-s001.pdf]

Table S1 The list of DNA probes used for FISH. The method and parameters of their preparation and labeling are indicated. N,M and L – variable parameters in the PCR without template program. N and M are increasing annealing temperatures, and L is extension temperature.

Probe	Source	Type of label	PCR conditions (if applicable), °C	Primers (if applicable)	FISH hybridization temperature, °C	temperature of FISH washes, °C
(AATAT)$_n$	nontemplate PCR	PCR	N=37<br>M=40<br>L=50	5′-(AATAT)$_4$-3′<br>5′-(ATATT)$_4$-3′	25	43
(AAGAG)$_n$	nontemplate PCR	PCR	N=55<br>M=60<br>L=72	L: 5′-(AAGAG)$_4$-3′<br>R: 5′-(CTCTT)$_4$-3′	37	43-60
(AAGAC)$_n$	nontemplate PCR	PCR	N=55<br>M=60<br>L=72	5′-(AAGAC)$_4$-3′<br>5′-(GTCTT)$_4$-3′	37	43-60
(AACAC)$_n$	nontemplate PCR	PCR	N=55<br>M=60<br>L=72	5′-(AACAC)$_4$-3′<br>5′-(GTGTT)$_4$-3′	37	43-60
(AAGAT)$_n$	nontemplate PCR	PCR	N=40<br>M=43<br>L=50	5′-(AAGAT)$_4$-3′<br>5′-(ATCTT)$_4$-3′	25	43-50
359het	PCR<br>The reference sequence for satell[illegible] 359het (1.688) was found in ref. [72]	PCR	annealing temperature=60	5′-TAGGGATCGTTAGCACTG[illegible]<br>5′-ACGAGCTCAGTGAGATAT[illegible]	37	43-60
(ACCGAGTACGGG)$_n$ (Dodeca)	nontemplate PCR	PCR	N=55<br>M=60<br>L=72	5′-(ACCGAGTACGGG)$_3$-3′<br>5′-(CCCGTACACGGT)$_3$-3′	37	43-60
(AATAACATAG)$_n$ (Prodsat)	nontemplate PCR	PCR	N=40<br>M=43<br>L=50	5′-(AATAACATAG)$_3$-3′<br>5′-(CTATGTTATT)$_3$-3′	37	43-50
Stellate	a 1.15-kb BglII fragment from *Stellate* repeats [71]	Klenow fragment labeling	---	---	37	43-60
28S rDNA	a 0.9-kb HindIII fragment of the 28[illegible] rRNA gene [70]	Klenow fragment labeling	---	---	37	43-60
rpl15	PCR	PCR	annealing	5′-TAAGTTGGTTGTGCATT[illegible]	37	43-60

			temperature=61	3′<br>5′-CTTTGCGTGCACGAAGT<br>[60]		
CG12406	PCR	PCR	annealing temperature=61	5′-<br>GGGGAAACTAGAAGGCCA<br>5′-TTGGCATTTTGATCGGA<br>[60]	37	43-60

Table S2. The read alignments number of the sequenced microdissection library against the database of repeated elements RepBase.

Repeat name	Reads count
ARS406_DM#Unknown	2511
SAR_DM#Satellite	1352
SAR2_DM#Satellite	1111
DOC#LINE/I-Jockey	81
DOC2_DM#LINE/I-Jockey	65
XDMR_DM#Unknown	58
Gypsy6_I#LTR/Gypsy	33
SAT-1_Dsim#Satellite	31
LSU-rRNA_Dme#rRNA	23
SSU-rRNA_Dme#rRNA	17
DM1731_I#LTR/Copia	16
FW_DM#LINE/I-Jockey	14
BLASTOPIA_I#LTR/Gypsy	13
R2_DM#LINE/R2	12
ROO_I#LTR/Pao	12
NOMAD_I#LTR/Gypsy	10
DM176_I#LTR/Gypsy	9
Copia_I#LTR/Copia	8
DM1731_LTR#LTR/Copia	8
XDMR#Unknown	8
BEL_I#LTR/Pao	7
HMSBEAGLE_I#LTR/Gypsy	7
MAX_I#LTR/Pao	7
BS2#LINE/I-Jockey	6
DM297_I#LTR/Gypsy	6
DNA4-1_DK#RC/Helitron	6
IS1#ARTEFACT	6
BATUMI_I#LTR/Pao	5
Gypsy6A_LTR#LTR/Gypsy	4
R1_DM#LINE/R1	4
R2_Dse#LINE/R2	4
STALKER4_I#LTR/Gypsy	4
G2_DM#LINE/I-Jockey	3
HOBO#DNA/hAT-hobo	3
SSU-rRNA_Hsa#rRNA	3
Homo6#DNA/hAT-Pegasus	2
NTS_DM#Satellite	2
BLOOD_I#LTR/Gypsy	1
DM176_LTR#LTR/Gypsy	1
Gypsy-11_DVir-LTR#LTR/Gypsy	1
Gypsy-37B_DWil-LTR#LTR/Gypsy	1
Gypsy-5_DGri-I#LTR/Gypsy	1
Gypsy-9_DBp-I#LTR/Gypsy	1
Helitron-1_DT#RC/Helitron	1
Helitron-N1_DBp#RC/Helitron	1
I-1_DBp#LINE/I	1

Jockey-1_DEr#LINE/I-Jockey	1
Mariner-2_DK#DNA/TcMar-Tc1	1
Mariner-3_Dan#DNA/TcMar-Tc1	1
MDG1_I#LTR/Gypsy	1
PARISa_Dan#DNA/TcMar-Tc1	1
R1_Dse#LINE/R1	1
R1_Dsi#LINE/R1	1
R1-17_Dwi#LINE/R1	1
R2_Dsi#LINE/R2	1
Stalker2_I#LTR/Gypsy	1
STALKER4_LTR#LTR/Gypsy	1
TRANSPAC_I#LTR/Gypsy	1
UVIR_DV#LINE/I-Jockey	1

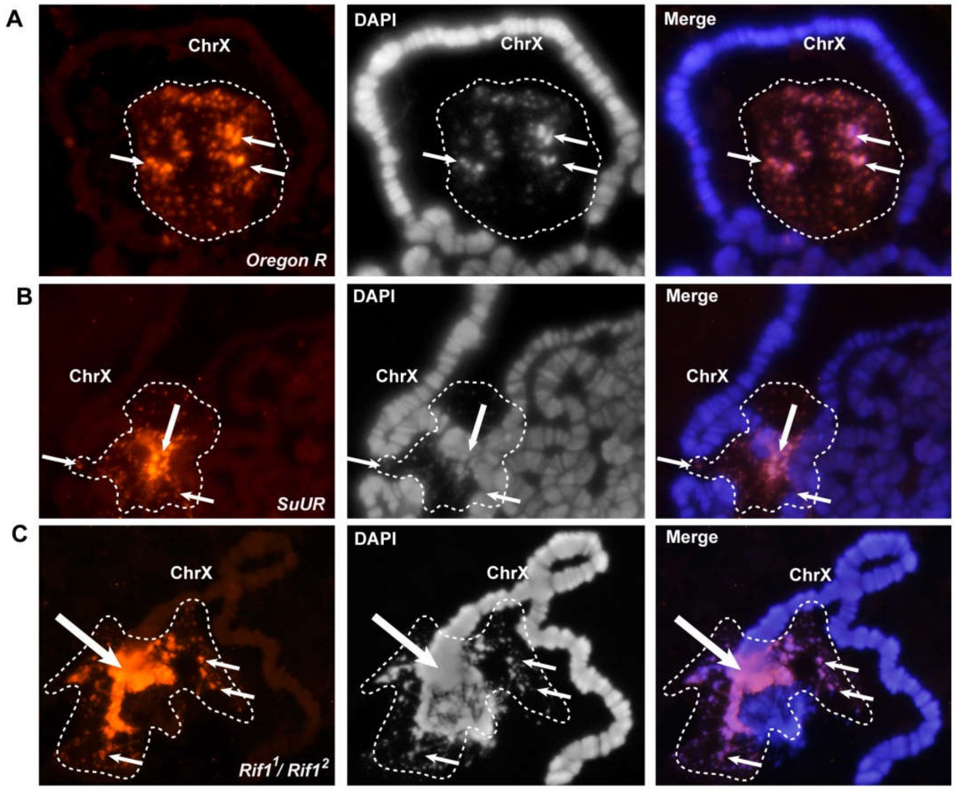

Figure S1. Effects of *SuUR* and *Rif1* on the nucleolus. *In situ* hybridization of the 28S rDNA probe on polytene chromosomes of Oregon R **(A)**, *SuUR*^{ES} **(B)**, and *Rif1*^{1}/*Rif1*^{2} **(C)** larvae. Dotted lines approximately outline the areas of nucleoli.

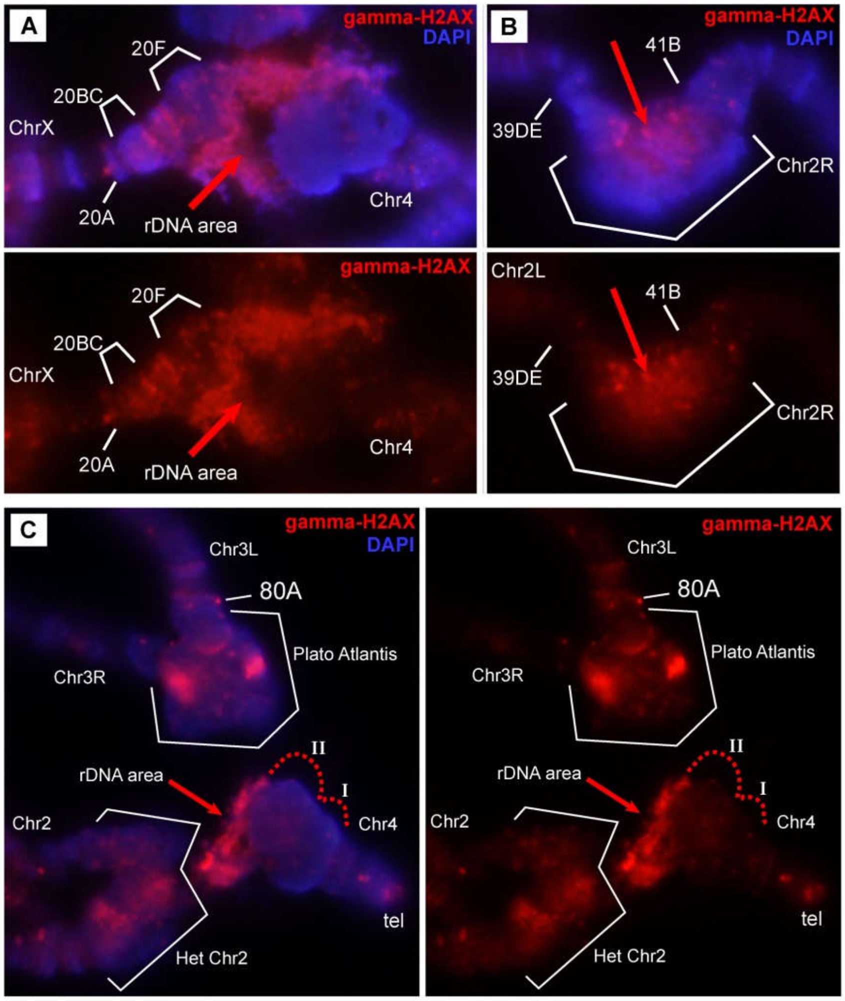

Figure S2. In the chromocenter of the *Rif1* mutants, underreplication zones remain. Localization of anti-γH2AX antibodies in the pericentromeric regions of joint chromosome X–4 **(A)**, chromosome 2 **(B)**, and all five chromosome arms **(C)** in *Rif1*^{2} mutants.

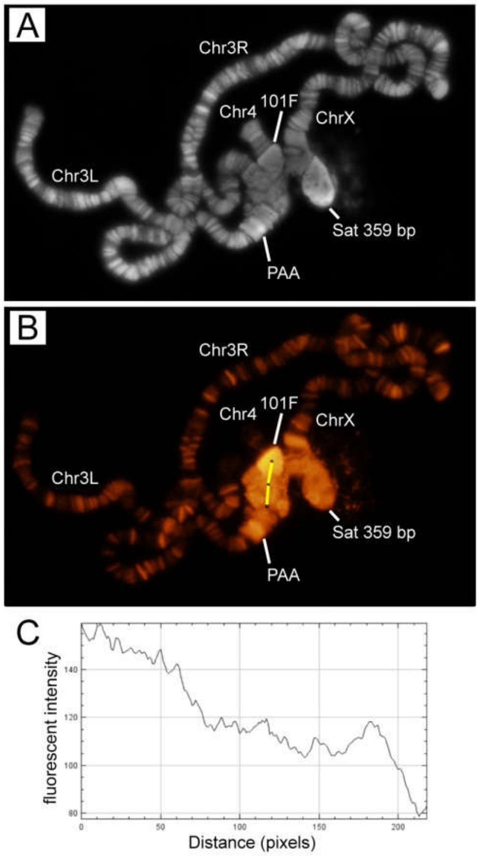

Figure S3. Quantification of fluorescence intensity after EdU detection along the heterochromatin at the MR–LR stage. The fluorescence intensity was determined along the yellow line passing through the bright heterochromatin block at the base of chromosome 4.

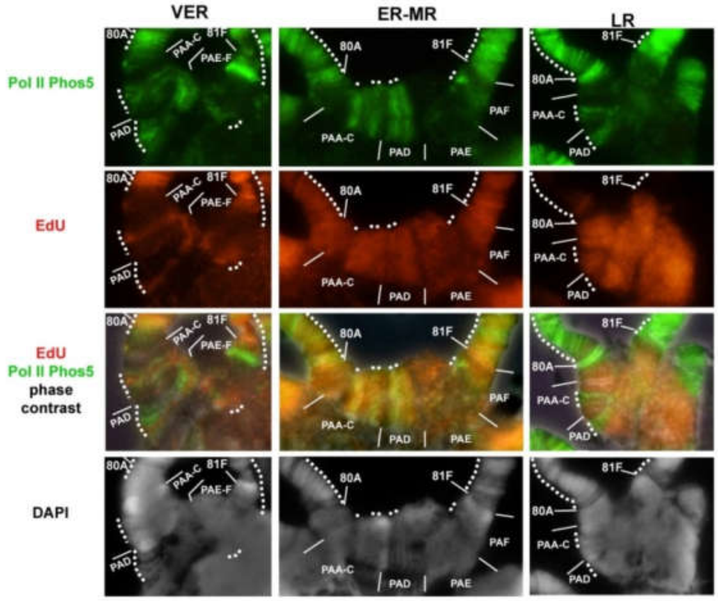

Figure S4. Interrelation of replication patterns in the pericentromeric region of chromosome 3 with the distribution of active transcription in polytene chromosomes of *Rif1* mutants. The left column: the VER stage of the S phase, the middle column: ER, and the right column: the LR stage. The figure illustrates the distribution of RNAPII Ser5P (first row), EdU (second row), an overlay of

red and green with a phase contrast image (3rd row), and DAPI staining (4th row). Dotted lines along the chromosome mark the areas where the signal was detected.

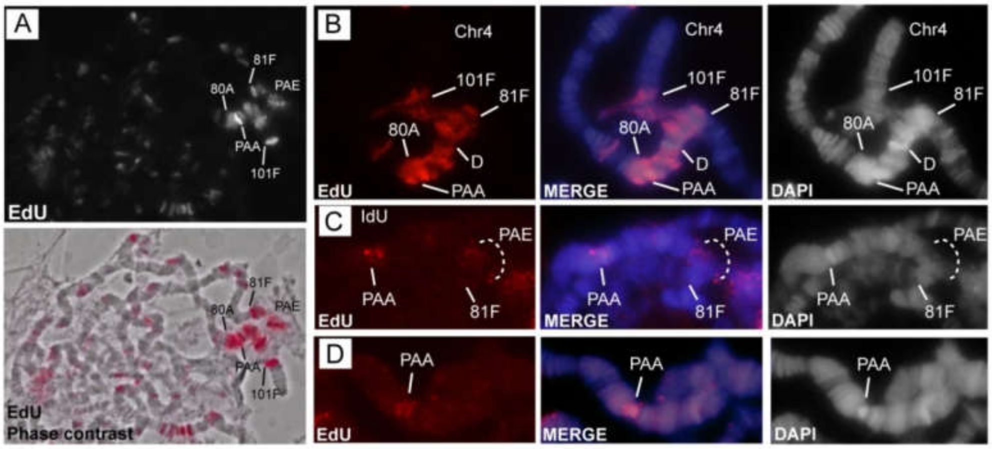

Figure S5. Late replication stages in the chromosomes of *SuUR^{ES} Su(var)3-9^{06}* mutants. (A) General view of the EdU incorporation pattern at the stage of LR. **(B–D)**. Three subsequent stages of very late replication.
